# Functional immune boosters; the herb or its dead microbiome? Antigenic TLR4 agonist MAMPs found in 65 medicinal roots and algae’s

**DOI:** 10.1016/j.jff.2023.105687

**Published:** 2023-07-29

**Authors:** E. Mazzio, A. Barnes, R. Badisa, G. Fierros-Romero, H. Williams, S. Council, K.F.A. Soliman

**Affiliations:** a Florida Agricultural and Mechanical University, College of Pharmacy and Pharmaceutical Sciences, Tallahassee, FL 32307, United States; b Florida Agricultural and Mechanical University, School of Environment, Tallahassee, FL 32307, United States; c John Gnabre Science Research Institute, Baltimore, MD 21224, United States

**Keywords:** Herbs, Edible microbiome, Medicinal microbiome, Bugs as drugs, Immune boosting

## Abstract

**Background:**

Humans have been consuming medicinal plants (as herbs/ spices) to combat illness for centuries while ascribing beneficial effects predominantly to the plant/phytochemical constituents, without recognizing the power of obligatory resident microorganism’ communities (MOCs) (live/dead bacteria, fungus, yeast, molds etc.) which remain after industrial microbial reduction methods. Very little is known about the taxonomic identity of residual antigenic microbial associated molecular patterns (MAMPs) debris in our botanical over the counter (OTC) products, which if present would be recognized as foreign (non-self) antigenic matter by host pattern recognition receptors (PRRs) provoking a host immune response; this the basis of vaccine adjuvants. As of today, only few research groups have removed the herbal MAMP biomass from herbs, all suggesting that immune activation may not be from the plant but rather its microbial biomass; a hypothesis we corroborate.

**Purpose:**

The purpose of this work was to conduct a high through put screening (HTPS) of over 2500 natural plants, OTC botanical supplements and phytochemicals to elucidate those with pro-inflammatory; toll like receptor 4 (TLR4) activating properties in macrophages.

**Study Design:**

The HTPS was conducted on RAW 264.7 cells vs. lipopolysaccharide (LPS) *E. coli* 0111:B4, testing *i*NOS / nitric oxide production $\left({\text{NO}}_{2}^{-}\right)$ as a perimeter endpoint. The data show not a single drug/chemical/ phytochemical and approximately 98 % of botanicals to be immune idle (not effective) with only 65 pro-inflammatory (hits) in a potency range of LPS. Method validation studies eliminated the possibility of false artifact or contamination, and results were cross verified through multiple vendors/ manufacturers/lot numbers by botanical species. Lead botanicals were evaluated for plant concentration of LPS, 1,3:1,6-β-glucan, 1,3:1,4-β-D-glucan and α-glucans; where the former paralleled strength *in vitro*. LPS was then removed from plants using high-capacity endotoxin poly lysine columns, where bioactivity of LPS null “plant” extracts were lost. The stability of *E.Coli* 0111:B4 in an acid stomach mimetic model was confirmed. Last, we conducted a reverse culture on aerobic plate counts (APCs) from select hits, with subsequent isolation of gram-negative bacteria (MacConkey agar). Cultures were 1) heat destroyed (retested/ confirming bioactivity) and 2) subject to taxonomical identification by genetic sequencing 18S, ITS1, 5.8 s, ITS2 28S, and 16S.

**Conclusion:**

The data show significant gram negative MAMP biomass dominance in A) *roots* (e.g. echinacea, yucca, burdock, stinging nettle, sarsaparilla, hydrangea, poke, madder, calamus, rhaponticum, pleurisy, aconite etc.) and B) *oceanic plants / algae’s* (e.g. bladderwrack, chlorella, spirulina, kelp, and “OTC Seamoss-blends“ (irish moss, bladderwrack, burdock root etc), as well as other random herbs (eg. corn silk, cleavers, watercress, cardamom seed, tribulus, duckweed, puffball, hordeum and pollen). The results show a dominance of gram negative microbes (e.g. *Klebsilla aerogenes*, *Pantoae agglomerans, Cronobacter sakazakii*), fungus (*Glomeracaea, Ascomycota, Irpex lacteus, Aureobasidium pullulans, Fibroporia albicans, Chlorociboria clavula, Aspergillus_sp JUC-2*), with black walnut hull, echinacea and burdock root also containing gram positive microbial strains (*Fontibacillus, Paenibacillus, Enterococcus gallinarum, Bromate-reducing bacterium B6* and various strains of *Clostridium*).

**Conclusion:**

This work brings attention to the existence of a functional immune bioactive herbal microbiome, independent from the plant. There is need to further this avenue of research, which should be carried out with consideration as to both positive or negative consequences arising from daily consumption of botanicals highly laden with bioactive MAMPS.

## 1. Introduction

Humans have been orally consuming medicinal plants for centuries to treat a myriad of human illnesses, many of which happen to be derived from the richest source of microbes found in the natural world; ocean and soil. While the plant microbiome has been extensively studied in relation to its growth, health, maturation, influence on environmental ecology (Berihu et al., 2023; Cook et al., 2023; Yin et al., 2023; Zhou et al., 2022) and food safety (Kazi et al., 2018; Wassermann et al., 2022), very little is known about the characterization or concentration of bioactive remnants of microbial debris in edible foods, including pathogenic after being intentionally destroyed. Specifically, there is limited knowledge about foods which would as a design by mother nature, contain high concentration of microbial/pathogen associated molecular patterns (MAMPs/PAMPS) with known capacity to modulate the immune system, this the fundamental basis for the development of vaccines, adjuvants and immunotherapies (Dong et al., 2014; Dubey et al., 2014; Nelson, 2022; Roeder et al., 2004; Rossi and Mastroeni, 2022; Zelechowska et al., 2022). Immune provocation is based on the ability to distinguish self from non-self, accomplished by host pattern recognition receptors (PRRs) that recognize foreign MAMPS (non-self) and include among others – the lipid A subregion of bacterial lipopolysaccharide (LPS) from gram negative rods. Concurrently, the human gut-immune axis, which plays a pivotal role in human immune resilience, houses cognate PRRs that are acted upon by these same MAMPS. However, despite advances in metagenomics, and the unveiling of taxonomy of live MOCS in raw foods (e.g. lettuce, bananas, olives, figs, sorghum etc. (Abdullaeva et al., 2021; Abera et al., 2022; Agarussi et al., 2022; Frohling et al., 2018; Higgins et al., 2018; Hong et al., 2023; Nakayasu et al., 2022; Soto-Giron et al., 2021)), research into the edible post processed heat destroyed microbiome and its effect on human immune activation is sparse.

Botanical microorganism community (MOC) biomass is a function of nature, which predicates its collective phenotype. Edible MAMPS in herbs, would not only mechanistically activate the Toll-like receptors including TLR4 such as lipoproteins, byglycans, acylated lipopeptides, lipoteichoic acid, peptidoglycans but also other TLRs (e.g. imidazoquinolines (TLR7) modulin, RNA from bacteria (TLR8) the synthetic dsRNA molecule polyinosinic-polycytidylic acid (poly-IC), glycolipids, fibrinogen/fibronectin, heat shock proteins, uric acid, flagellin (TLR5), ssRNA of microbial origin and unmethylated CpG rich DNA (TLR9) or for (TRL3) poly C derivatives). (Ehrchen et al., 2009; Galluzzi et al., 2012; Gulyas et al., 2022; Wolska et al., 2009a).

The diversity of plant microbiomes are a design of nature that would by logic be a function of; 1) the plants inherent bactericidal/ fungicidal properties (e.g. garlic (bulb), oregano, clove, thyme, green tea (leaves)) 2) the physical location of the plant/ part - from roots below soil (rhizome rich micro-biome)/ to leaves being above the soil (in direct exposure to sanitizing UV sunlight / pressurized rain wash off) 3) mitigating MOC changes during maturation to harvest 4) variation in circumscribing water systems, (storm,farm, industrial, oceanic) 5) wide array of MOC secondary metabolites produced 6) the adhesive nature of the plant enabling MOC concentration (e.g. sea alginates) 7) moisture and humidity (tropical to drought) 8) and post-harvest storage handling to modern advanced microbial reduction techniques employed.

Botanical food processing in civilized societies for over the counter (OTC) products assure the elimination of food borne pathogens (e.g. *Escherichia coli, Staphylococcus aureus, Salmonella enterica ssp typhimurium, Pseudomonas aeruginosa, Bacillus subtilis, Candida albicans and Aspergillus niger*). This requires use of broad based destructive processes ranging from dry/wet heat, fumigants (ethylene oxide/ propylene oxide) under vacuum or pressure, ultra-violet radiation, chlorine dioxide, ozone, CO_2_, or sophisticated oxidation techniques. (Jefri et al., 2022; Ma et al., 2022; Shi et al., 2021; Sholtes et al., 2016). Point of sale (POS) products sold as capsules, tablets or powders, therefore, contain not only the plant’s inherent content but also its defunct epiphytic microbiome, comprised of lack of viable pathogens, its dead microbiome and all other residual non-pathogenic live bacteria which remain as aerobic plate counts (APCs), reported as yeasts, molds and fungus in accordance to limits set by local pharmacopeia authorities, the world health organization (WHO) or Food Chemicals Codex monographs. (Becker et al., 2019; Jefri et al., 2022; Jovanovic et al., 2022; Ma et al., 2022; Shi et al., 2021; Sholtes et al., 2016).

While people have consumed plant microbiomes for thousands of years, only a small number of research teams - including our own – have suggested that the immune boosting (direct activation of innate or acquired immunity) effects of “specific herbs” may have little to do with the plant, but rather are inflicted by ingesting a decomposing bioactive microbiome. Pioneering research teams who’s work we confirm have shown MOC gram negative TLR4 activating MAMP biomass present in black walnut, echinacea root, ginseng root, alfalfa seeds (Pugh et al., 2008), astragalus root (Denzler et al., 2010; Koehler et al., 2020), angelica root; (Montenegro et al., 2015), and wheat (Kohchi et al., 2006; Mizuno and Soma, 1992; Nishizawa et al., 1992; Taniguchi et al., 2009). Meanwhile TLR4 MAMP agonists are being used in vaccines such as Cervarix^®^ (human papillomavirus vaccine) and Shringrix^®^ (herpes zoster) (Luchner et al., 2021) and there is greater acceptance of the idea that eating dead microbes can affect the immune system, with emergence of the field of *para*-probiotics (Vallejo-Cordoba et al., 2020). Similarly, the concept is well exemplified by the body of work on edible fungal beta-1,3/1,6-D-glucan derived from mushrooms or *Saccharomyces cerevisiae (yeast),* as well as spirulina (a gram negative rod) which can trigger anti-cancer immunity and provide greater response to infection through the gut-immune-systemic plexus (Al-Batshan et al., 2001; Albeituni et al., 2016; Geller et al., 2019; Li et al., 2010; Liu et al., 2015; Nelson et al., 2017; Ohno et al., 1988; Vetvicka, 2011; Vetvicka and Vetvickova, 2020; Xiang et al., 2012; Xin et al., 2022).

Mechanistically, once ingested, MAMPS activate immune cells (such as macrophages) and epithelial cells in gut-associated lymphoid tissues (GALT)/Peyer’s patches as well as subcomponent lamina propria via mechanisms involving TLRs, C-type lectin receptors, and NOD-like receptors (Crunkhorn, 2022; Patin et al., 2019). Once activated, MAMP/ PRRs transmit corresponding antigenic information via M cells to antigen presenting cells (APCs) which then transition toward mesenteric lymph (Mo et al., 2022) and systemic lymph via dendritic cells (Debnath et al., 2022; Iqbal et al., 2014; Paradia et al., 2022; Stander et al., 2022) triggering T cell clonal expansion. (Gallichan and Rosenthal, 1996; Smith et al., 2002; Woolverton et al., 1992). TLRs in the gut immune plexus have been identified on cell surfaces (TLR1, 2, 4, 5, 6) or / in (endosomal TLR3, 7, 8, 9) (Oblak and Jerala, 2011; Wolska et al., 2009b).

The data in this work, while suggesting a new area of plant medicine, apart from the plant itself, does not negate the incredible value of the plants phytochemical constituents having been reported extensively in the literature as having powerful “anti’ medicinal drug like properties” (anti-inflammatory, anti-pain (analgesic), anti-pyretic, anti-malarial, anti-bacterial, anti-protozoal, anti-parasitic, anti-oxidant, anti-viral etc.) (Aziman et al., 2014; Chen, 2022; Janthamala et al., 2021; Klein et al., 2013; Lu et al., 2019; Mazzio et al., 2016; Raposo et al., 2021; Romualdo et al., 2020; Sharifzadeh et al., 2016; Siddiqui et al., 2022; Silva et al., 2013; Zhang et al., 2021a; Zhang et al., 2021c; Zheng et al., 2018) In fact, these chemical attributes are the rationale behind extensive historical botanical / spice use in food preservation, (Chahal et al., 2021; Choi et al., 2017; El-Azzouny et al., 2018; Hosseinzadeh et al., 2014; Kim et al., 2020; Nabavi et al., 2015; Rodriguez-Garcia et al., 2016; Syahidah et al., 2017) and treatment of human infections (by lethal destruction to microbes) (Fahey et al., 2015; Howshigan et al., 2015; Tong et al., 2011; Zaidi et al., 2015). As of today, most all anti-cancer studies involving plants are focusing and reporting on phytochemicals acting on specific biotargets, as in blocking cell cycle, apoptosis, inducing / reducing transcription factors, oncogenes/ tumor suppressor proteins, receptors, cytokines and inflammatory signaling. (Al-Khayri et al., 2022; Shen et al., 2005). Contrarily and logistically, herbs that support systemic tumor immune boosting are cross parallel to those that include MAMPS, which are frequently heat/pH stable and may work synergistically with inherent plant compounds.

Here, in this work, we originally set out to conduct a routine high throughput screening of > 2000 botanicals of OTC nutraceuticals (sold through basic retail outlets worldwide) with intent to elucidate if any; could invoke macrophage polarization toward an M1 anti-cancer fighter phenotype (pro-inflammatory, anti-tumorigenic) which mechanistically could reverse an acquiescent M2 (anti-inflammatory,pro-tumorigenic) phenotype (Muller et al., 2017; Orange et al., 2016) beneficial to reawaken T-cell mediated adaptive host immune response to recognize self-malignant cells. (Chauhan and Kanwar, 2021; Evans et al., 2021; Galluzzi et al., 2012; Ghochikyan et al., 2014; Wang et al., 2023). The findings in our work, however, took a sharp turn after observing an unexplainable blatant inflammatory property beholden to specific herbs, on par with LPS. Here we establish a connection with previous research (sparse) suggesting that the plants MAMP laden microbiome and not the plant itself, appears to be directly aligned with activation of TLR4 signaling.

## Methods & Materials.

Hanks Balanced Salt Solution, (4-(2-hydroxyethyl)-1-piper-azineethanesulfonic acid) (HEPES), ethanol, sulfanilamide, 96 well plates, chemicals, general reagents, and supplies, were all purchased from either Sigma-Aldrich, (St Louis, MO, U.S.A.) or V.W.R. (Radnor, PA, U.S.A.). Natural products were purchased from Frontier Natural Products Co-op (Norway, IA, U.S.A.), Monterey Bay Spice Company (Watsonville, CA, U.S.A.), Mountain Rose Herbs (Eugene, OR, U.S.A.), Mayway Traditional Chinese Herbs (Oakland, CA, U.S.A.), Kalyx Natural Marketplace (Camden, NY, U.S.A.), Futureceuticals (Momence, IL, U.S. A.), Organic fruit vegetable market: New Leaf (Tallahassee, FL, U.S.A.), Florida Food Products Inc. (Eustis, FL, U.S.A.), Patel Brothers Indian Grocery (Tampa, FL, U.S.A.), Starwest Botanicals (Sacramento, CA, U.S. A.), Monteray, Bay Spice Co. (Watsonville, CA, U.S.A.), Banyan Botanicals (Williams, OR, U.S.A.), Bulk Supplements (Henderson, NV), Swanson Health Products (Fargo, ND, U.S.A.), Raven Moon Emporium (Rock Hill, SC, U.S.A.), Pure Organic (Solana Beach, CA, U.S.A.), NOW Foods, Supplements (Bloomingdale, IL, U.S.A.), American Standard Supplements (Ontario, CA), Carlyle Nutritional’s (Melville, NY, U.S.A.), Horbaach Health (Melville, NY, U.S.A.), Solaray (Salt Lake City, UT, U.S. A.), Terra Dolce Mycological Natural Products (Eugene, OR, U.S.A.), Daiwa Health Development (Gardena CA, U.S.A.), Earthborn Elements (Lindon, UT, U.S.A.), Natures Way (Green Bay, WI, U.S.A.), Nusapure (Los Angeles, CA, U.S.A.), T.N. Vitamins (Farmingdale, NY, U.S.A.), Maui Herbs (Kihei, Hawaii, U.S.A.), Vadik Herbs (Concord, California, U.S.A.), Arizona Nutritional Supplements (Chandler AZ, U.S.A.), Tame the Spirit Herbs (Mount Vernon,KY, U.S.A.), Terra Vita/ Terra Health (Sheridan, WY, U.S.A.), Western Botanicals (Spanish Fork, UT, U.S.A.), Toogood Botanic Co. (Richmond Hill, ON, Canada) and Mauwe / Maui Herbs (Maui, Hawaii, U.S.A.).

### 1.1. Cell culture

RAW 264.7 macrophages cells were purchased from American Type Culture Collection (ATCC) (Manassas, VA, U.S.A.). Cells were cultured in Dulbecco’s Modified Eagle Medium (DMEM) containing high glucose [4500 mg/L] supplemented with 7 % fetal bovine serum (FBS), 4 mM L-glutamine, and penicillin/streptomycin (100 U/ 0.1 mg/mL). Culture conditions were maintained at 37 °C in 5 % CO_2_ /atmosphere, and every 2–3 days, the media was replaced for sub-culture. For experiments, plating media consisted of DMEM (minus phenol red) [glucose 4500 mg/L], 5 % FBS, and penicillin/streptomycin (100 U/0.1 mg/mL). A stock solution of LPS from E. coli 0111: B4 was prepared in HBSS at 1 mg/mL and stored at –20 °C.

### 1.2. Library sample preparation

All natural chemicals, phytochemicals, and reference drugs were subject to stock preparation either being dissolved in dimethyl sulfoxide (DMSO) or ethanol [5–20 mg/mL], where all crude herbs in FAMUs botanical library were prepared in absolute ethanol [50 mg/mL] after being diced, macerated, and powdered, etc. then stored at –20 °C in the dark. All plants were cataloged by manufacturer, botanical and common names and given an internal I.D. number. All dilutions were prepared in sterile HBSS + 5 mM HEPES, adjusted to a pH of 7.4, ensuring solvent concentration of DMSO or absolute ethanol stayed at<0.5 % at working concentration.

### 1.3. Cell viability

Cell viability was assessed as a routine background measure, not a study endpoint, using resazurin [7-Hydroxy-3H-phenoxazin-3-one 10-oxide] (Alamar Blue) indicator dye. A working solution of resazurin was prepared in sterile HBSS minus phenol red (0.5 mg/mL), then added (15 % v/v) to each sample. Samples were returned to the incubator for 2–4 h, and the reduction of the dye by viable cells (to resorufin, a fluorescent compound) was quantitatively analyzed using a Synergy HTX multi-mode reader Bio-Tek Inc. (Winooski, VT, U.S.A.) with settings at [550 *nm* / 580 *nm*], [excitation/emission].

### 1.4. Hit Determination: Nitrite $\left({\text{NO}}_{2}^{-}\right)$ /indirect iNOS expression

Quantification of nitrite (NO2 − ) was determined using the Greiss reagent. The Greiss reagent was prepared by mixing an equal volume of 1.0 % sulfanilamide in 10 % phosphoric acid and 0.1 % N-(1-naphthyl)-ethylenediamine in deionized water, which was added directly to the cell supernatant post-treatment (experimental media consisting of DMEM - phenol red) and incubated at room temperature for 5 min. Controls and blanks were run simultaneously and subtracted from the final value to eliminate interference. Samples were analyzed at 540 nm on a Synergy H.T.X. multi-mode reader: Bio-Tek (Winooski, VT, USA).

## 2. Gram negative cell wall

### 2.0.1. Limulus Amebocyte Lysate (LAL) Testing

Concentrations of gram-negative microbial cell wall in herbs were quantified using the Pierce^™^ LAL chromogenic endotoxin quantitation kit (Thermofisher scientific, Waltham MA USA), which is based on a modified Limulus Amebocyte Lysate (LAL) reaction with LPS [Catalog number: A39552]. A major modification was needed due to the extreme high sensitivity and rapid signal saturation of this assay. In brief, reagent bottle 1 (LAL solution) and reagent bottle 2 (the chromogenic substrate solution) were diluted by 50 % using endotoxin-free water. Botanical samples (10 μg/mL) were prepared in endotoxin-free water, to which (half the recommended volume) 25 μl of LAL reagent bottle was added, and LPS standards (1 ng/mL to 87 ng/mL) were run on the same plate. After 3.5 min (at room temperature, no heat was applied), 50 μl of chromogen (half the recommended volume) was added, and the plates were read immediately at 1 plate read/ minute until the standard curve of LPS and samples were within the quantitative linear range for each herb. After the final read, stopping reaction was added. Samples were quantitatively analyzed using a Synergy HTX multi-mode reader Bio-Tek Inc. (Winooski, VT, U.S.A.) with settings at 405 *nm*. Note: we found cross-reactivity of this assay with β-glucans; therefore, we also conducted the glucan assays to ascertain false positives if any.

### 2.0.2. LPS -Biglycan ELISA

LPS presence was confirmed using the BioAssay^™^ ELISA for (Lip-oglycan, Endotoxin) manufactured by US Biologicals product number # 026552 and carried out according to the product manufacturer. Samples were read at 450 *nm* after adding the stopping reaction on an Bio-Tek Inc. (Winooski, VT, U.S.A.).

### 2.1. β-Glucan assay kit (Yeast and Mushroom)

Quantification of 1,3:1,6-β-glucan and α-glucan (yeast and mushroom) was determined using a kit purchased by Neogen/ Megazyme (Lansing, MI, U.S.A.) and carried out according to the manufacturer’s instructions (Assay procedure K-YBGL 02/21). Briefly, herbal ethanol extracts were solubilized /hydrolyzed in ice-cold sulfuric acid for 2 h, where glucan fragments were re hydrolyzed to glucose using a purified *exo*-1,3-β glucanase and β-glucosidase solution, followed by hydrolysis of α-Glucans hydrolyzed to D-glucose then measured with amyloglucosidase and invertase using the kits GOPOD reagent. Using a standard curve and blanks, samples were quantitatively analyzed using a Synergy HTX multi-mode reader Bio-Tek Inc. (Winooski, VT, U.S.A.) with settings at 510 *nm*.

### 2.2. β-Glucan assay kit (Mixed Linkage)

Quantification of 1,3:1,4-β-D-glucan was determined using a kit purchased by Neogen/ Megazyme (Lansing, MI, U.S.A.) and carried out according to the manufacturer’s instructions (Assay procedure A - K-BGLU 02/21) according to AACC Method 32–23.01/AOAC Method 995.16. Briefly, the following buffers were prepared to carry out the assay: sodium phosphate buffer [20 mM, pH 6.5], sodium acetate buffer (50 mM, pH 4.0), and sodium acetate buffer (200 mM, pH 4.0) Briefly, herbal extracts were diluted in sodium phosphate buffer, placed in boiling water for 60 s, and incubated at 100 °C for 2 min / 50 °C for 5 min prior to the addition of linchinase and further incubation at 1 h – 50 °C. After the addition of sodium acetate buffer, samples were equilibrated to room temp and centrifuged. β -glucosidase was added in 50 mM sodium acetate and then incubated at 50 °C for 10 min prior to the addition of the GOPOD reagent and subsequent quantification at 510 nm against a standard curve after subtracting blanks.

### 2.3. Endotoxin removal

Gram-negative cell wall was removed from herbal extracts using Pierces^™^ high-capacity endotoxin removal spin columns, 0.5 mL/ Catalog number: 88,274 (Thermofisher Scientific, Waltham MA, U.S.A.), and experiments were carried out in accordance with the manufacturer’s protocols. Columns were regenerated 4x for reuse and then discarded. Pre and post-column eluents were evaluated for macrophage activation against the standard curve of LPS (*E. coli* (0111:B4)) up to 5 μg/mL by measuring nitric oxide in RAW 264.7 macrophages at equal concentration set at 170 μg/mL.

### 2.4. Immunohistochemistry

Herbs were prepared (50 mg /mL EtOH) and centrifuged at 2000 x. Ethanol was removed, and the sample was reconstituted in methanol. Samples were vortexed, centrifuged, and the supernatant discarded, where residue was allowed to dry by evaporation overnight. Plant residue was reconstituted in PBS with 1 % casein blocking buffer for 20 min prior to the addition of 1:500 primary antibody (mouse anti - LPS antibody (Abcam, Cambridge, MA, U.S.A.) in microcentrifuge vials, then placed on a vertical rotator for 2 h at room temperature. After discarding the supernatant, the residue was washed 3X in PBS, centrifuging the residue and discarding the supernatant between washes. The secondary antibody secondary antibody 1:1000 (goat-anti-mouse Alexa 488) prepared in blocking buffer was added to tubes, and samples were placed on a rotator for 1 h at room temperature. After discard, the residue was washed 3x in PBS, centrifuging the residue and discarding supernatant between washes, with the last wash remaining mixed with the residue. Samples were then transferred to a 96-well plate and imaged using a Cytation 5 (Biotek) (Winooski, VT, U.S.A.).

### 2.5. Capsule to culture studies

Eight pro-inflammatory OTS/POS purchased in 2022 were subject to a starter culture. Samples were unpacked in a sterile working area/hood, and a starter culture of 50 mg/mL was grown at 34 °C for 1 week in RPMI media, no phenol red, with L-glutamine supplemented and 10 % FBS and without pen/strep. After one week, 40 μls of culture supernatant (absent residue) were transferred to 15 mL of media (same) and regrown for another two weeks at 34 °C. At this point, the cultures were highly concentrated and subject to microbiology studies and biological studies. For biological studies, cultures were boiled for 10 min and diluted in PBS with pen/strep and tested in a standard plating medium also containing pen/strep and examined for potency of macrophage activation.

### 2.6. Microbiology studies

Microbial cultures were subject to 10-fold serial dilutions in buffered peptone water. 2 mL aliquots taken from each dilution tube were transferred to 18 mL of nutrient broth (NB) and incubated at 37 °C for 24 h while shaking at 120 rpm. After 24 h, 10-fold serial dilutions were prepared from the NB medium and 100 μl of the dilutions were plated in duplicate on N.B. agar and MacConkey agar, a selective medium for Gram-negative bacteria and enteric bacteria and differentiated them based on lactose fermentation. The plates were incubated at 37 °C for 24 h (N.B. agar) or 48 h (McConkey agar). The colonies appearing on the plates were then counted, and the counts were recorded. The DNA of the isolates was extracted using DNeasy PowerLyzer ^®^, PowerSoil^®^ Kit QIAGEN according to manufacturer instructions for further sequencing.

### 2.7. Sequencing/species identification

Sequencing for both global colonies and gram-negative isolation was carried out using ribosomal 18S, ITS1, 5.8 s, ITS2 28S, and 16S rDNA sequence (1,542 bp) coding regions for species identification and community analysis by L.C. Sciences (Houston, Texas U.S.A.). In brief, the amplified library used for sequencing on the NovaSeq platform pairedend reads (2 × 250 bp). Were ITS2 primer:F (5′-GTGARTCATCGAATCTTTG-3′) R (5′-TCCTCCGCTTATTGATATGC-3′); ITS1 primer:F (5′-GAACCWGCGGARGGATCA-3′) R (5′-GCTGCGTTCTTCATCGATGC-3′). For 16S rDNA.The primers (341F/805R) designed to target the V3 and V4 regions of 16S rDNA generate an amplicon about 465 bp in size. The amplified library is sequenced on a NovaSeq platform with 250 bp pairedend reads mode (2 × 250 bp). Workflow involved DNA extraction, PCR amplification, Product purification, library preparation, and high through put sequencing. Bioinformatics analysis was carried out by raw data files in FASTQ format subject to reads merged by overlapping sequences, data quality control, and chimera filtering, resulting in high-quality clean data. DADA2 (Divisive Amplicon Denoising Algorithm) was used for dereplication (equivalent to 100 % similarity clustering), and generation of representative sequences at single-base resolution, greatly improving the data accuracy and taxonomy resolution. The core function of DADA2 is denoising, leading to the construction of Operational Taxonomic Units-like table, namely ASVs (Amplicon Sequence Variants) table. The resultant characteristic representative ASV sequences are key for downstream analyses, including diversity, taxonomy, and differential analyses. Taxonomy: required use of SILVA (Release 138, https://www.arb-silva.de/documentation/release-138/), NT-16S database, RDA and Unite database for taxonomy, confidence level set to be > 0.7. We use for taxonomy, confidence level set to be > 0.7. Based on the ASV annotation results, the abundance, and differential results at different levels, ie., Domain, Phylum, Class, Order, Family, Genus, and Species, can be determined. Data is reported as Species.

### 2.8. Stomach simulation

A stomach model simulation was developed to test the ability of hydrochloric acid (HCl) to inactivate the biological activity of LPS. HCl was prepared from 0.007 N to 2 N in sterile water containing 5 mg/ml phenol red, along with a blank. pH was acquired for each acid solution on day 1, 2 and 3 to ensure stability. On day 3, after equilibration with ambient air, neutralization strategies were pilot tested on 500 ul of each HCL concentration with drop by drop addition of 0.05, 0.5 and 2 N NaOH grossly demarcated first by visual targeting using phenol red indicator dye. Volumes of added NaOH were recorded, and end pH was checked using an elite pH spear. All solutions were neutral.

After the above neutralization methods were validated - each concentration of HCl was placed in a 15 mL conical tube filled to 8 mL. LPS was added to all HCl serial dilutions at 5ug/ml. After adding LPS/ the LPS/HCl solutions were neutralized at Time 0 (immediately) 1, 2 and 4 h using the technique above and stored at 4°C. All samples were then tested for macrophage activation *in vitro* as previous described.

## 3. Data analysis

General statistical analysis was performed using Graph Pad Prism (version 3.0; Graph Pad Software Inc. San Diego, CA, U.S.A.) with the significance of the difference between the groups assessed using a one-way ANOVA then, followed by Tukey post hoc means comparison test or a Student’s *t*-test. IC_50_s were determined by regression analysis using ATT Bioquest analytic tools.

## 4. Results

### 4.1. HTPS setup

**Fig. 1.**
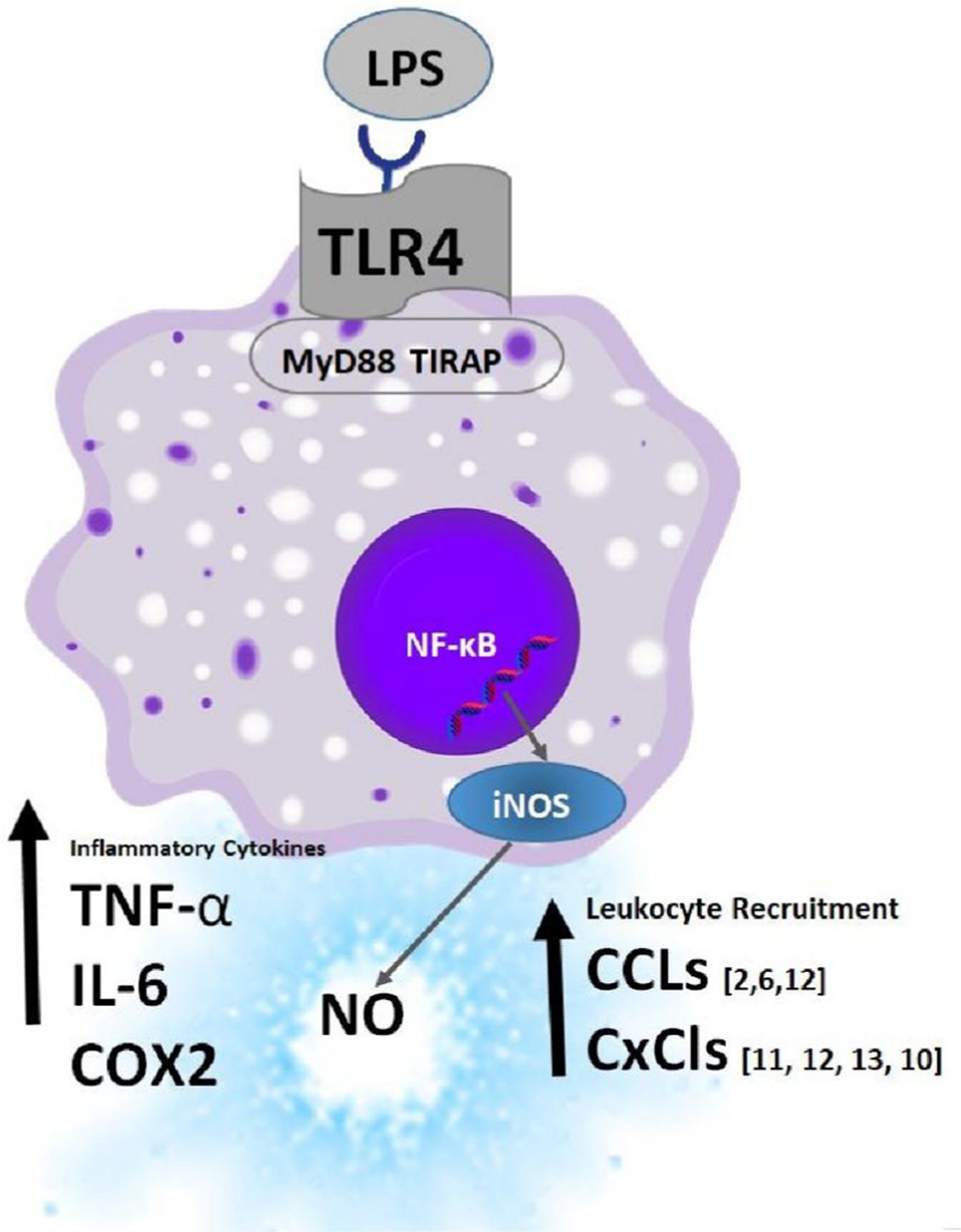
Hit Description; Rapid screening of RAW 264.7 macrophages by LPS E. Coli 0111:B4 by quantifying the production of nitric oxide (NO). LPS activates the pattern recognition receptor TLR4, invoking recruitment of myeloid differentiation primary response gene 88 (MyD88) to its cytosolic domain and adaptor protein TIRAP (T.I.R. adaptor protein), initiating translocation of nuclear factor kappa-light-chain enhancer of activated B cells (NF-κB) into the nucleus from the cytoplasm where induction of genes (Sakai et al., 2017) encoding chemokines involved in leukocyte recruitment (CCL2, CCL6, CCL12, CXCL10, CXCL11, CXCL12, CXCL13), inflammatory cytokines (TNF –α, IL-6), iNOS, thereby making the easily quantifiable release of nitric oxide (NO) by colorimetric detection using the Griess Reagent a target for high throughput screening.

The pro-inflammatory effects of an in-house natural product and botanical library (2532 compounds) were determined by measuring $\left({\text{NO}}_{2}^{-}\right)$ production (endpoint) in RAW 264.7 macrophages [1 × 10] cells /mL] in 96 well plates using (LPS *E.Coli* 0111:B4) as a positive control, and cell solvent blank as a negative control. The mechanism by which LPS acts on PRRs / TLR4 is well known where it can trigger endocytosis, initiating recruitment of myeloid differentiation primary response gene 88 (MyD88) to the cytosolic domain. (Kolanowski et al., 2014; Sakai et al., 2017) (Fig. 1). Subsequently, mitogen activated protein kinase signaling elicits translocation of nuclear factor kappa-light-chain enhancer of activated B cells (NF-κB) is directed into the nucleus where it upregulates a host of genes involved with leukocyte recruitment (CCL2,CCL6,CCL12, CXCL10, CXCL11,CXCL12,CXCL13), a pro-inflammatory response (IFN-γ, TNF –α, IL-6 or Type I interferons IFN- α and IFN-β), and iNOS induction and production of $\left({\text{NO}}_{2}^{-}\right)$. (Luchner et al., 2021).

LPS/ TLR4 agonists are not only used in modern vaccines as adjuvants, to link innate to adaptive immune response (Luchner et al., 2021) but also trigger anti-tumor signaling in cells bearing its receptor with the most studied being tumor associated macrophages (TAM) M1 anti-cancer fighter phenotype (pro-inflammatory, anti-tumorigenic) which reverses the acquiescent M2 (anti-inflammatory pro-tumorigenic and stimulated by IL-4, IL-13, IL-10 phenotype) (Muller et al., 2017; Orange et al., 2016). Administration of LPS, in diverse tumor models leads to activation of M1 TAMS and a host systemic reawakening of the T-cell mediated adaptive host immune response to recognize self-malignant cells (Chauhan and Kanwar, 2021; Evans et al., 2021; Galluzzi et al., 2012; Ghochikyan et al., 2014; Wang et al., 2023).

For rapid screening, $\left({\text{NO}}_{2}^{-}\right)$ is easily detected as an indicator of macrophage activation, using a visible chromogen (Griess Reagent) suitable for high throughput screenings.

**Fig. 2.**
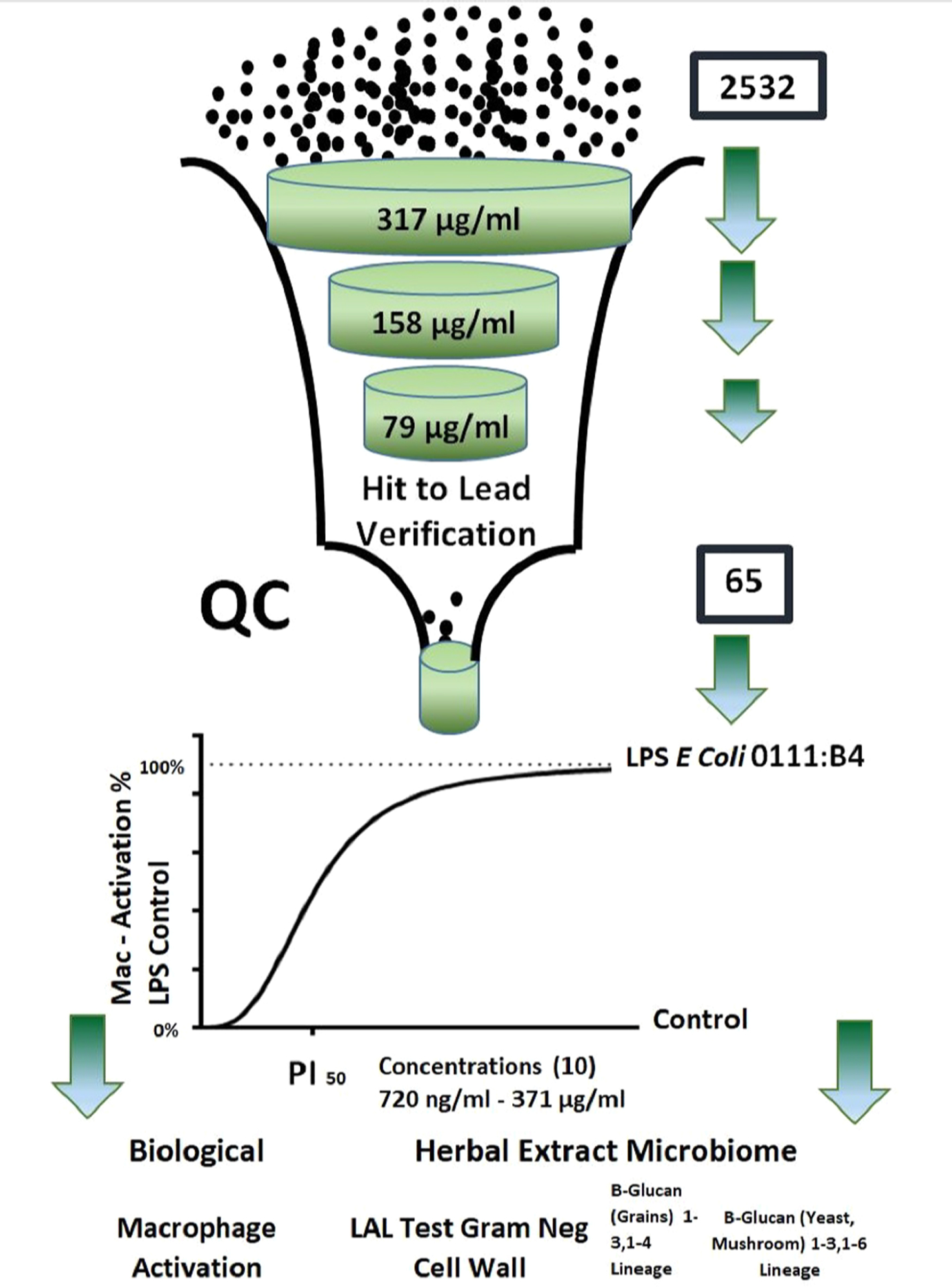
Study Design: A high throughput screening library comprised of 2532 compounds [366 drugs, 193 phytochemicals, and 1973 OTC /POS botanical ethanol extracts] were evaluated for NO production in macrophages vs. LPS E. coli 0111: B4 positive controls (1 μg/mL). Hits and toxic compounds from Tier 1 (T1) tested at 317 μg/mL were tested at subsequent lower concentrations (T2 and T3), and toxic compounds at lower concentrations if needed. 65/2532 botanicals were Q.C. validated as pro-inflammatory, none of which were phytochemicals or drugs, all absent of interfering artifact or contamination, being predominantly repeatable across vendors (Table 1). These 65 were then subject to a dose–response potency test (Pro-inflammatory 50 concentration PI50) (conc. range: 0.72–371 μg /mL) established from an LPS standard curve (1 ng/ mL-1000 ng/mL) run simultaneously with all samples, having the LPS (Max) value at 500 nm set at 100 % LPS control. All 65 herbal extracts were then tested for the presence of gram-negative cell wall and β-glucans. (Table 1).

The HTPS libraries were cataloged from dry material intake, ascribed an ID, with data records kept on vendor, lot number, botanical identity and cultivation region, with most all plant based botanical products and OTC products accompanied by a certificate of analysis. All stock solutions were prepared for drugs/ phytochemicals in either ethanol or DMSO, and all herbs were macerated, powdered and placed in sterilizing ethanol at 50 mg/ml. All compounds were prepared in stock plates containing endotoxin free HBSS, and tested *in vitro*, under sterile conditions. All compounds were tested in macrophages cultured in media with pen-strep (note: lethal to gram positive, and gram-negative bacteria). A Tier 1 screening; 2532 compounds (all herbs tested at 317 μg /mL) (Fig. 2) either rendered an absence of response, a pro-inflammatory response or a toxic response, the latter tested in subsequently lower concentrated Tiers. All hits and toxic compounds were rescreened through a series of lower concentrations starting at Tier 2 (158 μg/mL), Tier 3 (79 μg/mL) and lower, for macrophage activation.

**Fig. 3.**
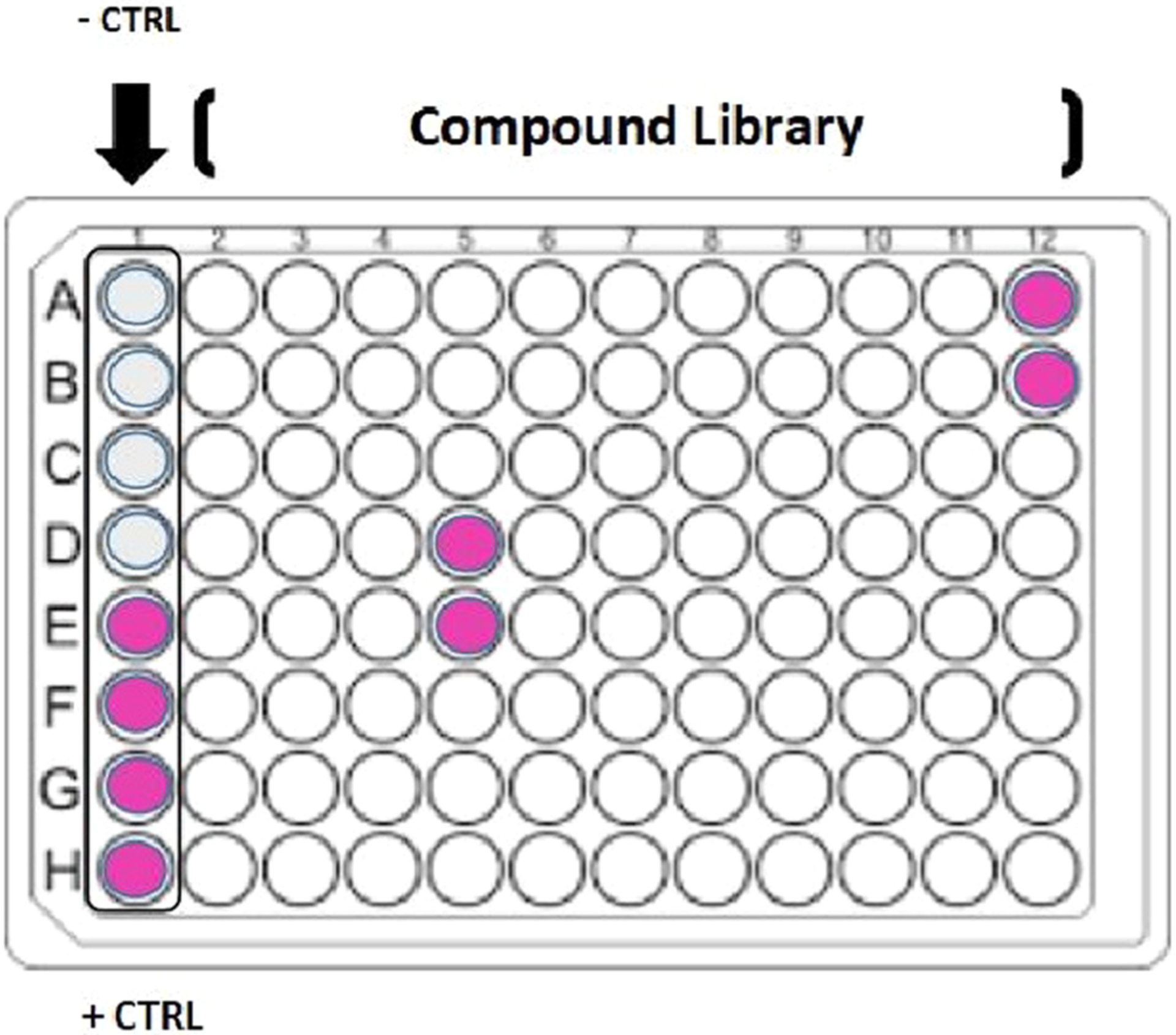
HTS plate setup. Screenings were conducted in 96 well plates, with positive and negative controls in column 1 (A-H).

The standard screening layout in 96 well plates contained four positive/ four negative controls in Column 1 with samples as shown in Fig. 3. The data showed a lack of macrophage activation by all drugs and phytochemicals, with hits only in botanicals - comparably potent to LPS capacity to elicit $\left({\text{NO}}_{2}^{-}\right)$ production. All hits were subject to validation testing to eliminate plausible artifacts, including 1) reagent control reactions (matrix blanks with the Griess Reagent) 2) consistency across various vendors and lot numbers (product specific) and 3) strength of bio-active response by pro-inflammatory 50 (PI_50_) concentrations vs. 100 % LPS control and 4) quantification of gram-negative microbial LPS and various β-glucans (Table 1). LPS standard curves for dose - time response were required for every follow-up study in macrophages to maintain quantification by regression matched batch controls, with measurements made on the linear portion of standard curves, relative to cell density.

Table 1 provides basic information on the botanical identity of “hits” by common name, botanical name, the number of vendors tested for the same botanical species, ratio of hits/ vendor tested ( % hit ratio), (immune potency of the herb) as (PI_50_) and concentrations of gram-negative cell wall (endotoxin unit (EU) or (ug) and β-glucans by lineage (g/100 g) w/w.

### 4.2. Column removal of gram-negative cell wall

**Fig. 4.**
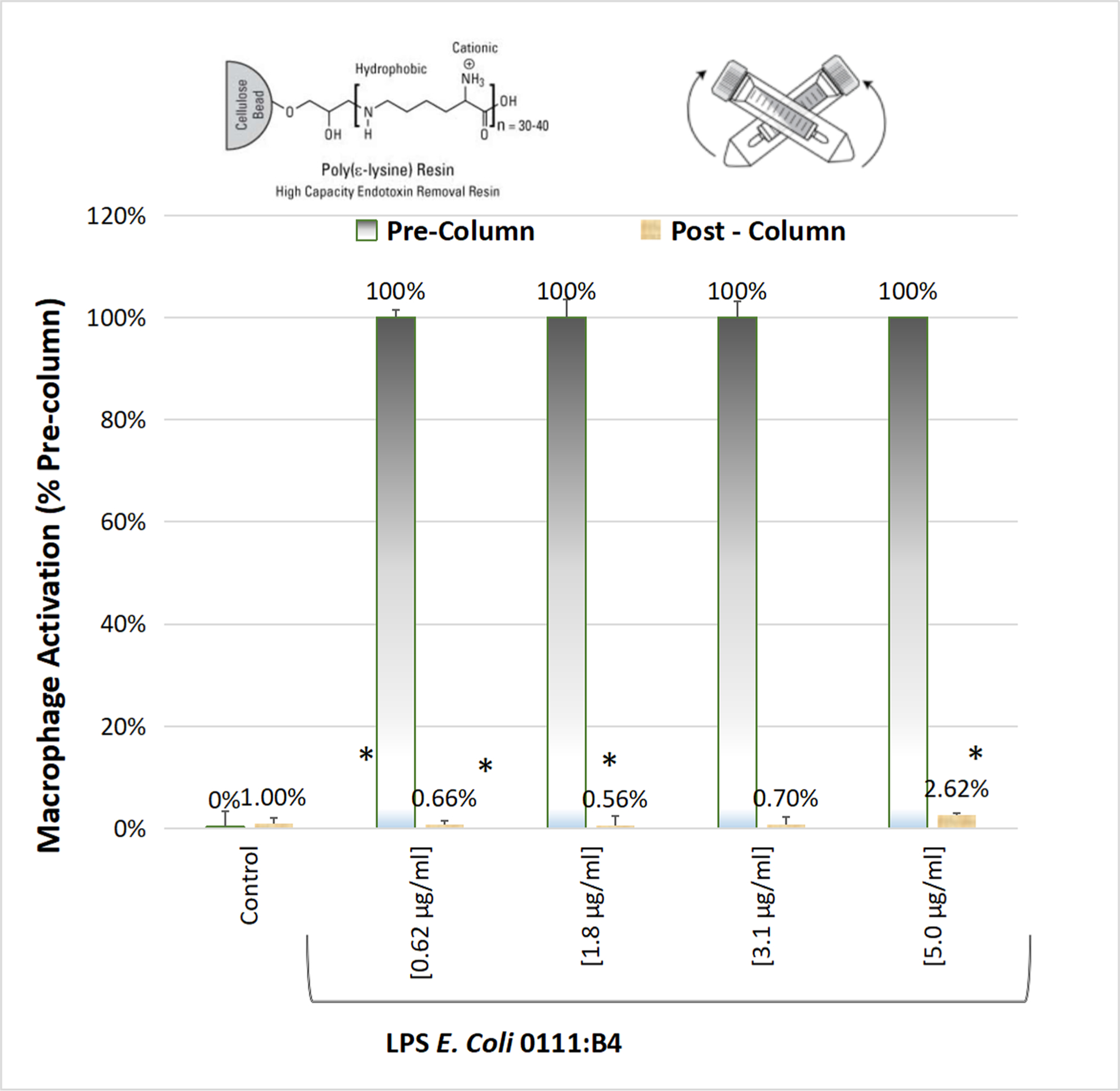
Endotoxin Removal Method validation: A method validation was run prior to removing LPS from the 65 herbs. The data represents Pre (100 %) vs Post (% of Pre) by macrophage activation response, measured by iNOS / NO2 production at equal concentrations. LPS (0.62 to 5ug/mL) was tested where the data is expressed as the Mean ± S.E.M., n = 2 column separations. Significant differences for pre vs. post macrophage activation was determined using a *t*-test *P < 0.01.

LPS was removed from plants using high-capacity endotoxin removal columns. Method validation studies were first performed to ensure efficacy of colum ns to remove LPS *E.Coli* 0111:B4 up to 5ug/mL (Fig. 4). Bioactivity of post vs pre column eluents for all samples are provided in Table 2. Endotoxin removal was effective in reversing macrophage activation for all herbs-except for black walnut hull and marine plants. After testing eluants for LPS, it was believed that marine plants either had a greater affinity for MAMPs than the polylysine stationary column packing, or its presence exceeded capacity. This will require further investigation as marine kelps and seaweeds contain adhesive alginates, “seaweed gums,” a main ingredient in microbiological growth agar. The ability of alginates to seed bacterial colonies could result in a natural high-capacity microbial reservoir inherent to oceanic plants. None the less LPS was visualized by centrifuge immunohistochemistry in kelp and bladderwrack residue (Fig. 5A) and confirmed by ELISA (Fig. 5B).

### 4.3. Gram negative LPS pH stability – stomach model simulation

**Fig. 6.**
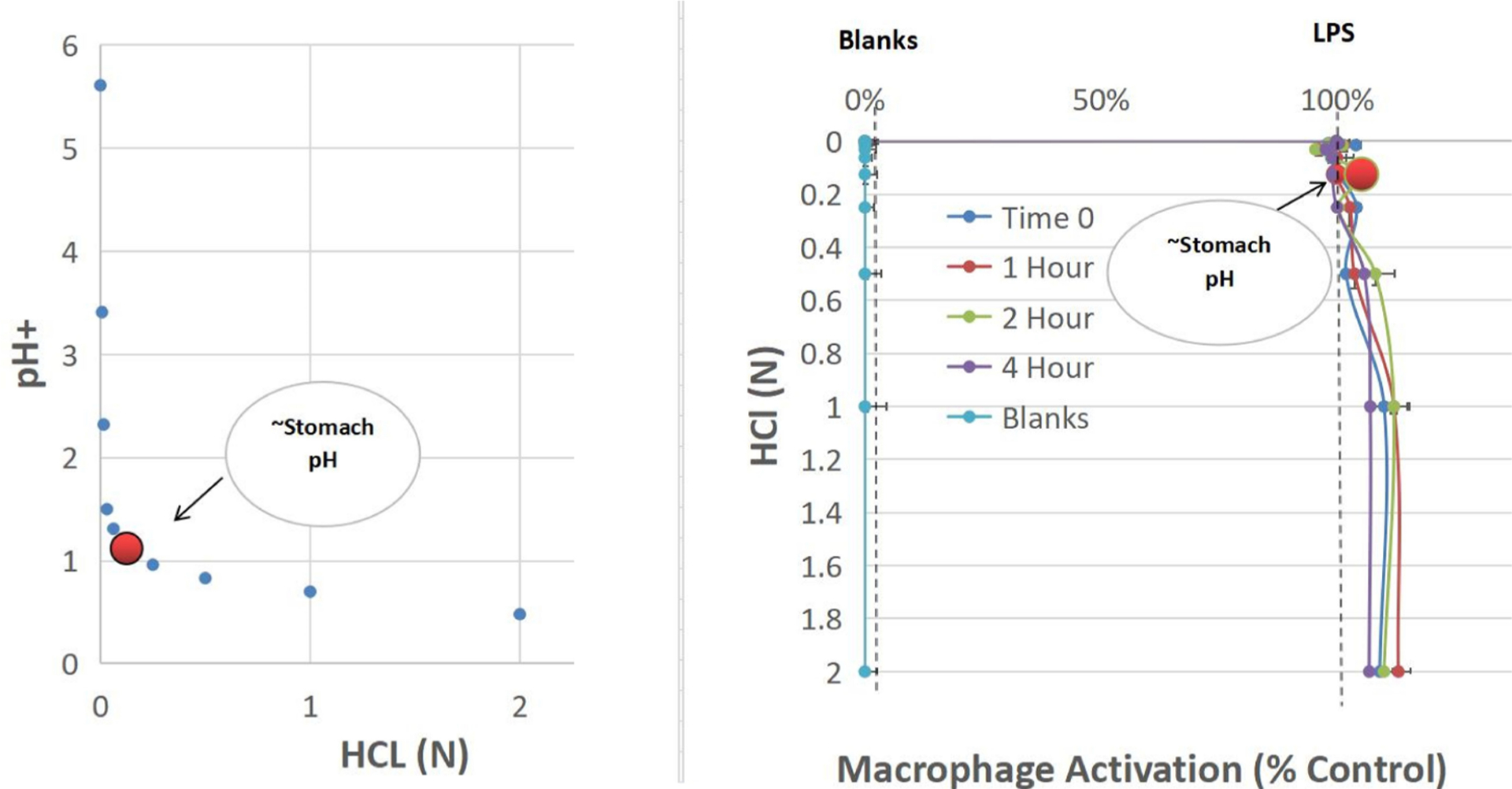
LPS stability – using a stomach acidity model. LPS was incubated for 0 to 4 h in varying concentrations of hydrochloric acid, also evaluated for pH (left panel). After neutralization, solutions were tested for biological activity (right panel) on RAW 264.7 macrophages for nitric oxide production vs. LPS control. The data represents macrophage activation (% LPS control) for varying acidity and exposure time and is presented as the Mean ± S.E.M., n = 3. There was no significant loss of biological activity well beyond stomach acidity, with no effect for 4 h of incubation in 2 N HCL at < 0.5 pH.

One of the main questions to the concept of this work, would be do edible MAMPS withstand stomach digest or acidity. A basic stomach model was created by examining a wide range of H + acid concentrations above and below the known concentration of HCl released from parietal cells in the stomach (0.16 N, pH 1–2) - range from 0.007 N to 2 N HCL and for 0–4 h (Fig. 6). LPS 5ug/ml was placed in varying acid solutions for 0–4 h, neutralized with NaOH, and retested in RAW 264.7 macrophages for bioactivity vs LPS control. We found no negative effects of LPS upon incubation with HCl up to 2 N, and<0.5 pH with an exposure time of 4 h.

### 4.4. Gram positive vs. Gram negative TLR4 activation

**Fig. 7.**
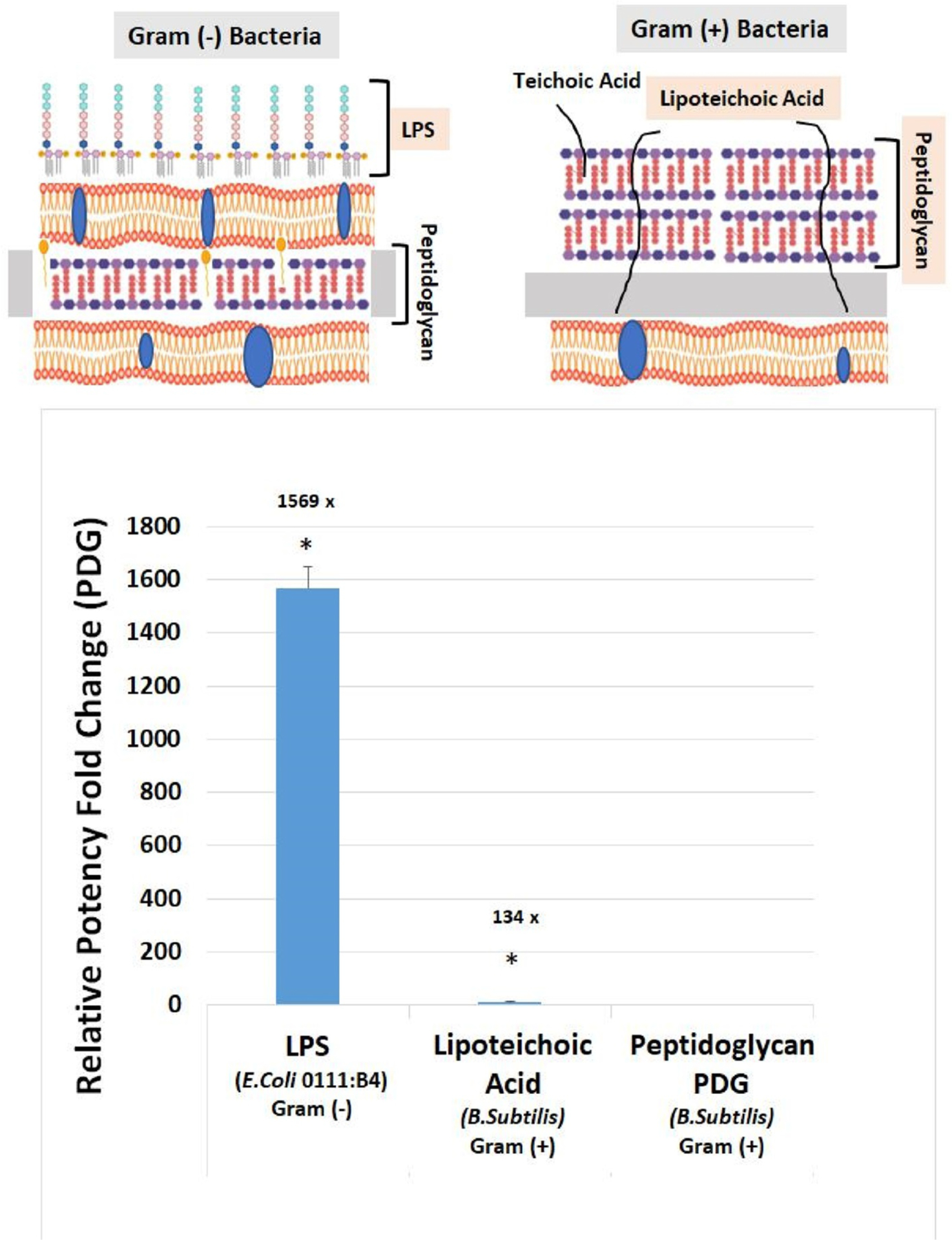
Relative potency of gram-negative vs. gram-positive cell wall to activate RAW 264.7 macrophages. The data is expressed as the Mean ± S.E.M., n = 4 with significant differences from peptidoglycan (PDG) (gram-positive cell wall from B. Subtilis) for both Lipoteichoic Acid (gram-positive cell wall from B. Subtilis) and LPS (gram-negative cell wall from E.Coli) determined using a *t*-test *P < 0.01.

Comparable macrophage activity was assessed in response to gram-positive (B. Subtilis), gram-negative (*E. Coli*) (Fig. 7) and lipoteichoic acid. The latter was 134X more potent than peptidoglycan (PDG) and LPS was 1569X stronger than PDG.

### 4.5. Capsule to culture - residual live reverse culture

**Fig. 8.**
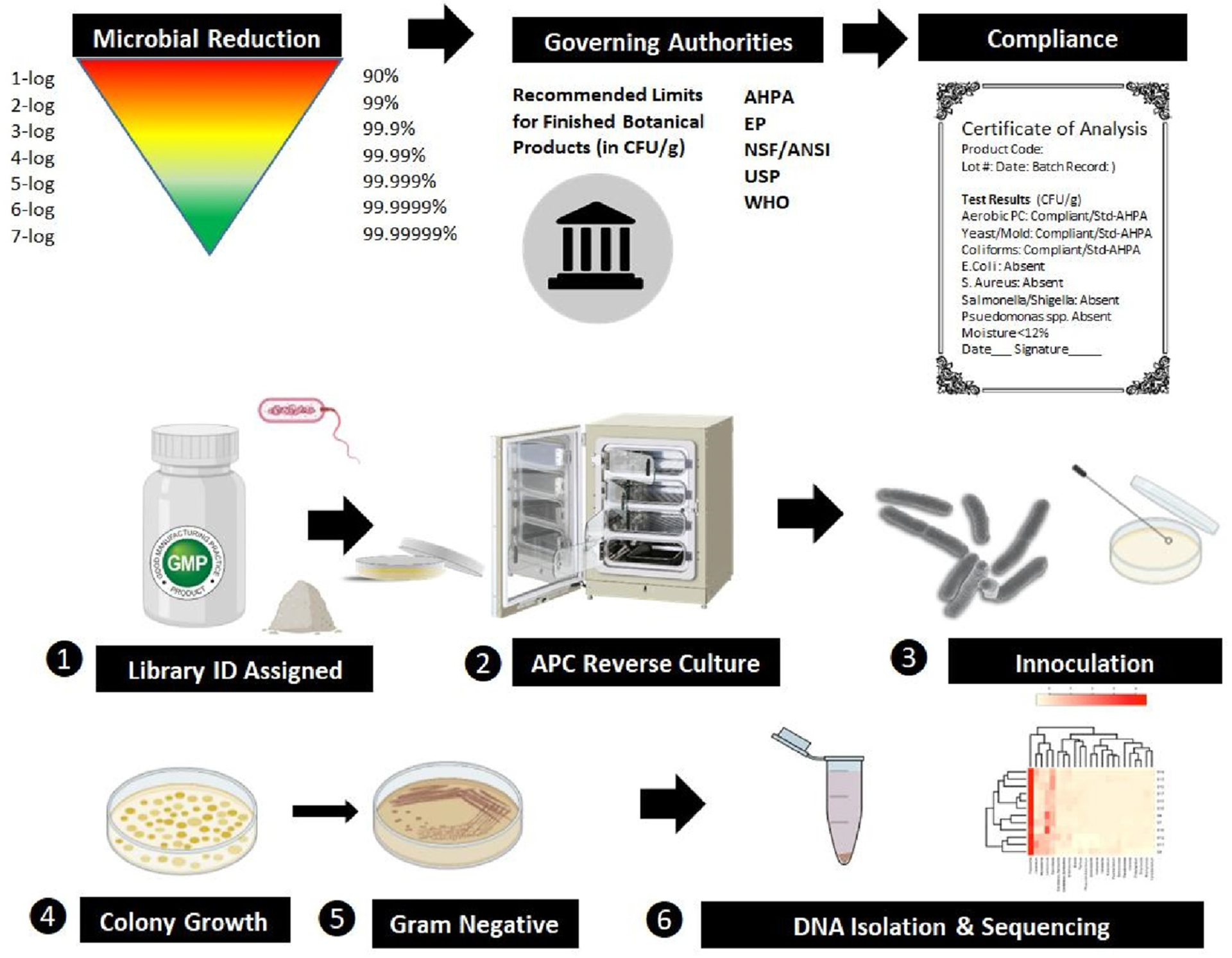
Capsule to Culture Schematic: Prior to FAMU OTC/POS botanical library cataloging, manufacturers are required to test each lot/ batch using recommendations for finished products by a regulating authority, including the world health organization (WHO), NSF/ANSI standards, various pharmacopeia’s (European (E. P.) or United States (U.S.P.). A certificate of analysis is issued with information on passing criteria for residual live aerobic plate counts, coliform, yeast, molds, and absence of viable pathogens or toxic mold products. For capsule studies (1), upon receipt of the OTC/POS product, a library I.D. is assigned designating manufacturer, lot number, and botanical identity. (2) We chose 8 inflammatory herbs and tested these by reverse culture in sterile media (no / pen strep) supplemented with F.B.S. at 34◦ C. After three weeks of growth and subculture, samples were transferred to agar (3) to observe colony growth (4) and gram-negative presence (5). DNA from cultures were isolated, and sequencing to identify micro-organism communities (Table 3).

All herbal botanical products pass a 3rd party testing (chain of custody) (Fig. 8) documented by a certificate of analysis for aerobic plate counts, (by the most stringent recommendation), not to exceed 10^4^ colony forming units (CFU)/ gram, total yeasts and molds at < 10^3^ CFU/ gram, total coliforms < 10^2^ CFU/g with the absence of E. Coli / 10 g. Reverse culture of a few immune stimulating botanicals sold at OTC starting at [50 mg/mL herb] was carried out at 34 °C for one week, then diluted and sub-cultured for two weeks at 34 °C, then boiled and retested on macrophages where potency was magnified. Cultures were grown on standard agar and subsequently colony isolation using MacConkey agar for gram-negative presence. DNA was isolated and further subject to 16S amplicon and metagenomics shotgun sequencing to survey bacterial communities (Table 3).

**Fig. 9.**
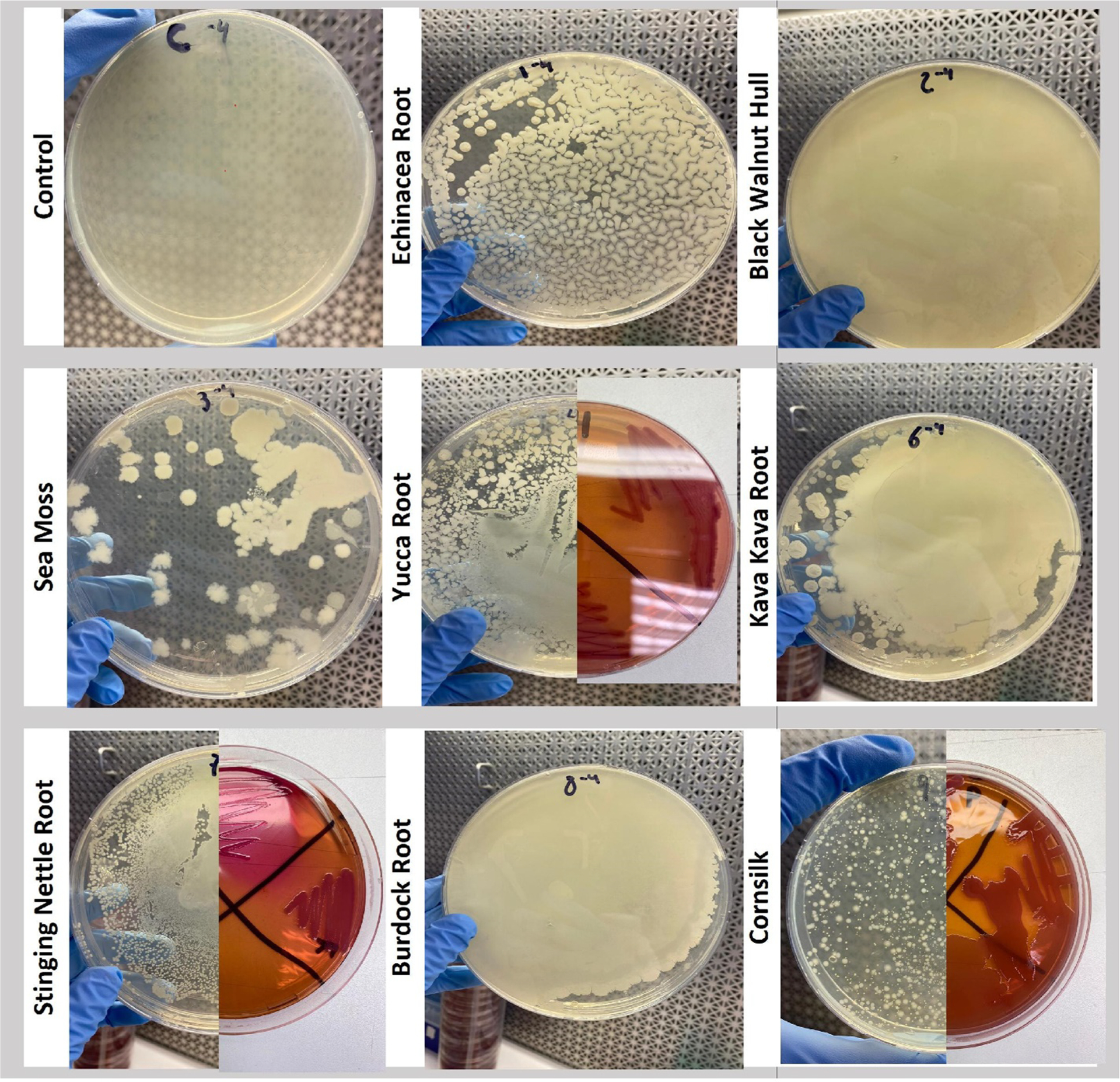
Representative macroscopic images of agar plates incubated at 37 ◦C for 24 h in nutrient broth agar (NB agar) or 48 h (McConkey agar) isolated from yucca root, stinging nettle, and cornsilk. A) Microbial aerobic growth on NB agar (whole or left half) and B) Gram-negative bacteria growth on MacConkey agar if present (right half) are shown.

**Fig. 10.**
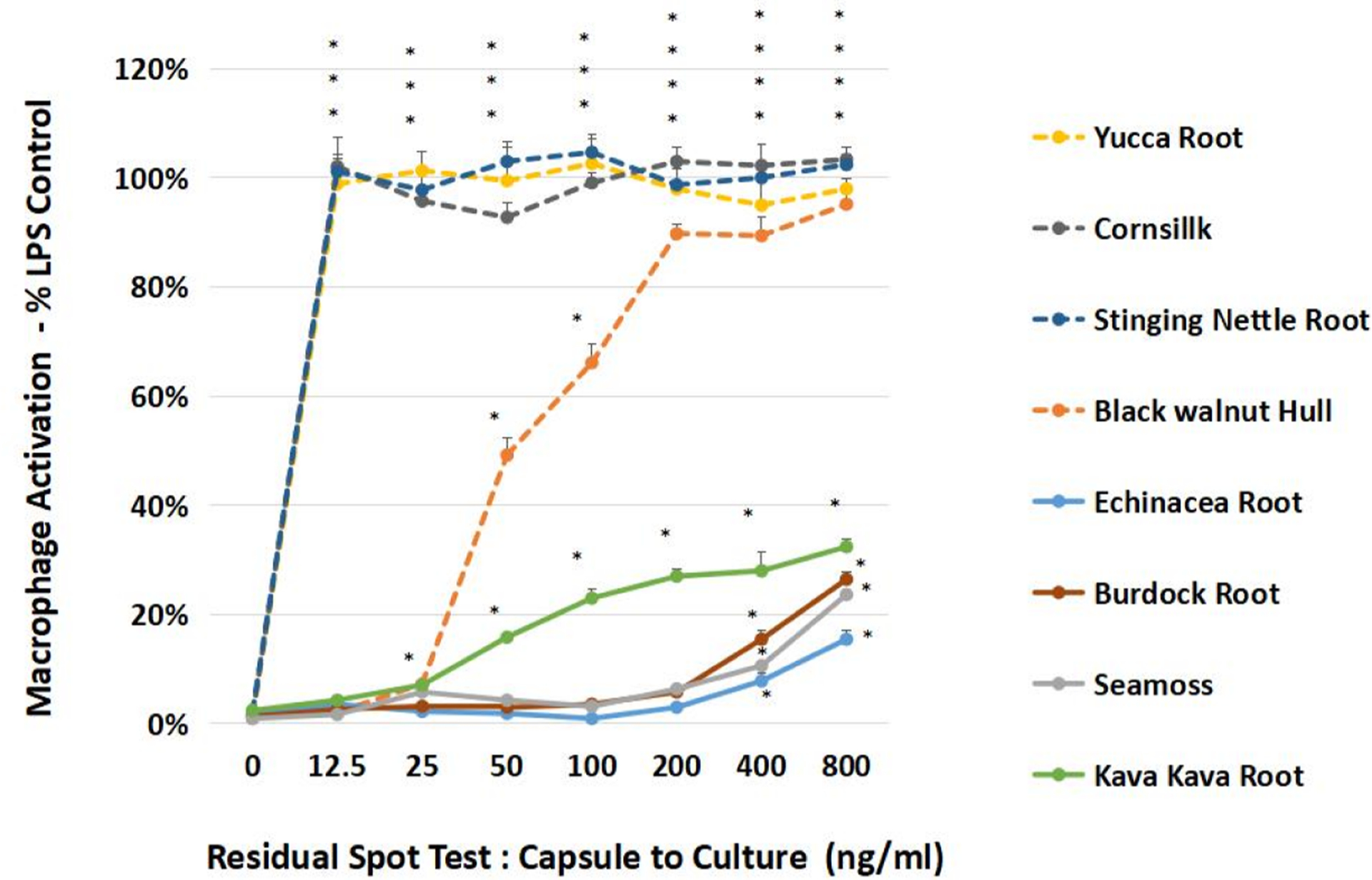
Bioactivity of heat inactivated reverse cultured plant microbiomes. The data are presented as macrophage activation % LPS E.Coli 0111:B4, represented by the Mean ± S.E.M., n-4. Significant differences from the control were determined by a one-way ANOVA, with post hoc Tukey test, *P < 0.05.

Table 3 provides a list of microbial communities by reverse culture, which alone invoked macrophage activation, after boiling (Fig. 10). The data here are very rudimentary where we spot-tested herbs such as stinging nettle, corn silk, and yucca root which tested positive for gram-negative cell wall (Fig. 9), where TLR4 macrophage activation to produce $\left({\text{NO}}_{2}^{-}\right)$[below 12 ng /mL] occurred greatest in LPS laden herbs in addition to black walnut hull (Fig. 10). The most dominant microorganisms in capsule-to-culture reverse culture were as follows:

- **Stinging Nettle root [Gram negative/fungus]** (Klebseilla aerogenes, Pantoea agglomerans, Gomeracea, Ascomycota, Cryptococcus sp., Dothiedeomycetes sp.),
- **Yucca root [Gram negatives/fungus]** (Cronobacter sakazakii, Irpex lacteus, Chlorociboria clavula, Gomeracea, Ascomycota, Crytptococcus sp. Taphrina pruni).
- **Corn Silk [Gram negative/fungus/spiromonas]** (Pantoea agglomerans, Klebseilla aerogenes, Enterobacter cloacae, Enterobacter sp. Aspergillus_sp, and colpodella angusta),
- **Black Walnut Hull [Gram Positive/ Fungus]** (Fontibacillus, Paenibacillus, Auerobasidium pullulans, Aspeprgillus sp, Malassezia sympodialis, Irpex lacteus).
- **Echinacea Root [Gram Positive/ Fungus]** (Gomeraceae, bromate-reducing bacterium B6, Clostridium sensu stricto 7,18 and 3_unclassified) Lachnotalea, Lysinibacillus).
- **Kava Kava Root [Gram Positive/ Fungus]** (Paenibacillus, Glomeraceae, Fibroporia albicans, Bacillus thermoamylovorans, Malassezia restricta, Fontibacillus, Auricularia polytricha, Chlorociboria clavula).
- **Seamoss [Gram Positive/ Fungus ]** (Clostridium_sp KOPRI80182, Gomeraceae, Clostridium sensu stricto 1, Aspergillus sp. JUC-2, bromate reducing bacterium B6,Bjerkandera sp. CPCC 480726).

Future studies will have to be conducted on many fronts to further investigate the nature of the therapeutic immune modulating plant microbiome.

## 5. Discussion

Regarding plant-based medicinal research, as seemingly correct, the plant itself and its constituent phytochemicals have received most of the attention over the past century. Whereas, the plant microbiome has received most attention in the study of agriculture in pursuit of understanding MOC roles in ecological balance of soil, plant, water, and air systems (Diedhiou et al., 2016; Shahid and Khan, 2022), with meager research as to impact on human health. Food based MOC debris is by no means ubiquitous in concentration or taxonomy, and it is ever present in all processed foods, beverages, and even bottled drinking water (tap, bottled, mineral, and natural water), to exception of that treated by reverse osmosis. (Tischner et al., 2021; Vasudevan et al., 2021; Zhou et al., 2021). Given that our entire diet from the natural world involves ingestion of MOCS (Irshad et al., 2022; Jiang et al., 2021; Soto-Giron et al., 2021) some which are intentionally added (generally recognized as safe (GRAS)) for fermentation (Chen et al., 2019; Tang et al., 2021; Voidarou et al., 2020; Wang et al., 2017; Yan et al., 2022) there is a pressing need to assess, these bioactive entities in herbs and spices (in particular) for impact on human immune function (F et al., 2022; Neuzil-Bunesova et al., 2022; Ssepuuya et al., 2019).

In this work, we focus on botanicals and related OTC supplements used by consumers world wide which are certified to be devoid of ***live*** food borne pathogens (Finger et al., 2021; Karam et al., 2021; Levy et al., 2015; Willis et al., 2015) and contain threshold limits for live aerobic bacteria counts (APC) (Feroz et al., 2016; Garbowska et al., 2015), yeasts, and molds. However, this only describes industry compliance and according to reports on actual shelf products (herbs/spices) pulled, 92.1 % contain live, unidentified bacteria, and 5.3 % have counts that are beyond acceptable APC levels (>100,000 CFU/g), the majority of which are unidentified fungi, mold, or yeasts (Dlugaszewska et al., 2019). Due to the antibacterial properties of the plant/parts, such as those found in thyme, there is great disparitiy between low CFUs/g (eg. 2,100 CFU/g) or oregano, 1,500 CFU/g, to extremely high counts found in paprika (100,000 CFU/g) and pepper (820,000 CFU/g) (Schwab et al., 1982). Again our results confirmed these findings showing extremely high MAMP loads found in paprika for 7 / 8 manufacturers examined globally, and low to no bioactive MAMP content in thyme, oregano or other antibacterial leaf based products.

Our findings, also align with research reporting high levels of gram-negative LPS found in wheat (Mizuno and Soma, 1992; Nishizawa et al., 1992), black walnut, echinacea root, ginseng root, alfalfa seed (Pugh et al., 2008), astragalus root (Denzler et al., 2010; Koehler et al., 2020), the immune boosting herb Juzen-taiho-to (Montenegro et al., 2015) and kelp. (Inagawa et al., 1992a) LPS in wheat flour (LPSw) from *Pantoea agglomerans* reportedly bestows ameliorative effects on diverse health ailments such as diabetes (Iguchi et al., 1992), ulcer (Inagawa et al., 1992b) and hyperlipidemia (Kobayashi et al., 2018; Mizobuchi et al., 2021; Okutomi et al., 1992).

While some suggest LPS to be therapeutic, the term LPS as endotoxin has a negative connotation. In fact, “endotoxin” is a single derogatory term used to describe cell wall from all gram negative species, despite concentration, or its association with deadly sepsis, health benefits when consumed as spirulina LPS (gram negative rod) or as a therapeutic immune booster vaccine adjuvant. (Luchner et al., 2021) In 1856, Peter Ludvig Panum first referred to LPS as “putrid poison”, which was later termed endotoxin by Richard Friedrich Johannes Pfeiffer in association with Cholera, followed by its chemical extraction from Salmonella typhimurium in 1933 (Kolmos, 2006; Ogawa et al., 2007) to elucidation of its structure. LPS contains 1) a terminal O-antigen hydrophilic polysaccharides (serotype strain) 2) a leading lipid A region (anchor) linked to 3) a core region by 3-deoxy-d-*manno*-2-octulosonic acid (Kdo) with L- *glycero*-D-*manno*-heptose (l,d-Hep) and hexoses and hexosamines (Kong et al., 2004; Mazgaeen and Gurung, 2020). In 1952, Westphal and Lüderitz developed a hot phenol method for LPS purification / extraction from gram negative bacteria and in 1954, identified lipid A as the immune active endotoxic substance, (Osborn, 2019) later classified as a PAMP capable of activating human TLR4 PPR receptors. (Gauthier et al., 2022; Mazgaeen and Gurung, 2020) The ability of LPS to interact with PRRs is directly related to its structure and can differ in its endotoxic potential based on the fatty acid composition of the lipid A structure and /or the O-antigens (lacking) rough vs. rich in O-antigen (smooth). See Review: (Mazgaeen and Gurung, 2020). Meanwhile, humans have been ingesting LPS (e.g. spirulina (Arthrospira platensis) a gram negative rod ) (Besednova et al., 1979) for over five centuries to boost human immune resilience.

### 5.1. Bacterial LPS and immune response

The use of bacterial MAMPS (including LPS) as an immune therapy to treat human cancers has an extensive history, dating back to the late 1800s, where cancer patients experienced curative remission after contracting acute febrile infection with Streptococcus pyogenes (Carlson et al., 2020; Okuyama et al., 2017). There after, cancers were treated with an “attenuated” mixture of Streptococcus pyogenes (gram positive peptidoglycan) with *Serratia marcescens* (gram negative LPS) evoking curative remission in approximately 50 % of mesodermal embryonic origin cancers (sarcoma, lymphoma, leukemia, kidney, ovarian etc (Hobohm, 2001; Hoption Cann et al., 2003; McCarthy, 2006). Today, immune-suppressive medicines are main line drugs often used to treat cancer, making infection a leading cause of death of cancer patients. Meanwhile the scientific community is attempting to renovate rediscovery in anti-tumor immunotherapies.

Nevertheless, the study of LPS and its role in cancer yields contradiction within the literature. However, data is consistent when aligned to use of models, end points, and route of administration. On the one hand, studies using LPS in immunocompetent, immune-system-intact animals show inoculation regimes are quite effective in reducing tumor burden (ovarian, liver, glioma etc.) (Ignacio et al., 2019; Okuyama et al., 2017; Won et al., 2003), coinciding with significant changes in boosting of immune function (Chicoine et al., 2001; Chicoine et al., 2007). LPS has also been shown to lessen the side effects of antibiotics and chemotherapies ((e.g., LPS or gram-negative microbes (e. g., *Alistipes shahii*) (Soma and Inagawa, 2015) and oral administration e. g. (*Pantoea agglomerans*) establishes complete recovery and remission in 62 % of patients (Morishima and Inagawa, 2016). Likewise , clinical trials using synthetic LPS Lipid A subunits show tumor reduction (such as ONO-4007, DT 5461), not withstanding tolerance which becomes a limiting therapeutic issue (Matsumoto et al., 1998; Matsushita et al., 2003). Alternatively, research establashing LPS as pro-tumor largely employimmune deficient models such as nude mice, cancer cells without an immune co-culture, or by general expression profile that shows TLR4 to be abundant in malignant tumor tissue relative to normal.

### 5.2. Marine plants and gram negative LPS

The oceans are teeming with plants that have been invaded by microbial communities that share common living quarters, being essential for plant growth (Ramirez-Puebla et al., 2022), preservation of aquatic ecosystems, natural recycling, plant immunity (Weinberger, 2007) and with greater accrual in aged plants (Lemay et al., 2021). The data in this study are in direct alignment with existing marine biology studies such as that on Chlorella (*Chlorella vulgaris*), Spirulina, Dulse (*Palmaria palmate*), Irish Moss (*Chondrus crispus*), Seamoss (combination of Irish Moss and Bladderwrack (*Fucus vesiculosus*) all showing a high presence of gram negative microbes (and thus LPS). (Miranda et al., 2022; Weigel and Pfister, 2021). Other reports show high gram negative presence in the sea kelps (*P. scouleri Laminaria setchellii, N. luetkeana, Chondrus crispus, Laminaria ochroleuca, Palmaria palmate* etc.) *Granulosicoccus antarcticus*, *hellea balneolensis* (Miranda et al., 2022; Weigel and Pfister, 2021) which reside amongst hundreds of bacterial strains rich in *Bacillaceae* and *Enterobacteriaceae*.(Del Olmo et al., 2018; Picon et al., 2021) The same is true for coastal ocean sea grasses, with gram-negative biofilms, dominated by *Granulosicoccus* sp. *(Gammaproteobacteria) bacteroidetes and Alphaproteobacteria* and coincide with efficacy of brown seaweeds such as bladderwrack (*Fucus vesiculosus*) which also bestow tumor suppressive effects (Gupta et al., 2020), on ovarian (Bae et al., 2020) estrogen-dependent cancers (Zhang et al., 2016) head and neck (Blaszczak et al., 2018), thyroid (Shen et al., 2017) and pancreatic cancers (Geisen et al., 2015). These studies all show a pattern by which medicinal use of sea plants can evoke stimulation of natural killer (NK) cells (Zhang et al., 2021b) or greater peripheral blood mononuclear cell counts (Park et al., 2021). These heavily laden microbial reservoirs have been consumed for centuries, with a plethora of recent research showing the same to instill anti-tumor immunity (Ishiguro et al., 2020; Skjanes et al., 2021) in various cancers including colon, breast (Yuan and Walsh, 2006), hormone-related (Skibola et al., 2005) and thyroid (Rosen et al., 2014). For instance, chlorella attenuates tumor growth in animal models (Kubatka et al., 2015) which coincides with increased natural killer cell (NK) activity, macrophage activation and proliferative lymphocytic capacity (Kim et al., 2022). Similar, spirulina, a gram-negative microbe renders LPS that increases phagocytosis and cytokine-mediated inducible nitric oxide production, while enhancing immunity against cancer and infection (Al-Batshan et al., 2001). These investigations are generally in line with reports that certain sea kelps and seaweeds will reduce the risk of developing malignancies of the biliary tract, the prostate, or the breast (Nelson et al., 2017), prostate (Ohno et al., 1988) or breast cancers, reports which represent the foundation of traditional Japanese cultural dietary practices.

## Roots

The results reported in this work are also in alignment with agricultural studies pertaining to the root bed in plants showing heavy presence of certain microbes “seed to rhizo-micro-biomes”(Neemisha et al., 2022; Orozco-Mosqueda et al., 2022) which dominate plant growth (Chang et al., 2022; Zhang et al., 2020), rigor, fitness, and crop yield (Abdullaeva et al., 2021) Koprivova and Kopriva, 2022; Li et al., 2022). Microbial communities residing in roots are diverse, some including non – pathogenic *B. cereus* group, and spp of *Enterobacter, Pseudomonas, Cronobacter*, with those close to water having the greatest diversity (Sun et al., 2019). In this study, we demonstrate that the roots with the highest MAMP content were: Tou Weng: English Pulsatilla Root (*Pulsatillae Radix*), Black Cohosh Root (*Cimicifuga racemose*), Burdock Root (*Arctium lappa*), Butchers Broom Root (*Ruscus aculeatus*), Calamus Root (*Acorus calamus*), Cao Wu (Zhi): English Wild Aconite Root (*Aconitum kusnezoffii*), Dandelion Root (*Taraxacum officinale*), Echinacea Root (*Echinacea purpurea*), Feng Fang Root (*Radix Saposhnikoviae*) Hydrangea Root (*Hydrangea arborescens*), Kava Kava Root (*Piper methysticum*), Lou Lu/Rhaponticum Root (*Rhaponticum Uniflorum*), Nettle Root (*Urtica dioica*), Pleurisy Root (*Asclepias tuberosa*), Poke Root (*Phytolacca Americana*), Qian Cao/Madder Root (*Radix Rubiae*), Sasparilla (*Smilax medica*), *Sheng Jiang Pi (Zingiber Officinale*), Soapwort root (*Saponasia officinalis*) and Suma Root (*Pfaffia paniculata*).

The study of plant lignans and phytochemicals, which are thought to be the cause of these effects, has dominated research on the therapeutic benefits of roots up until this point. While effects of these products on “ immune boosting’ have been reported extensively (e.g Burdock root (Chan et al., 2011; He et al., 2018; Suzuki et al., 2002; Wang and Yang, 1993), Essiac (1998) Kava Kava Root (Minh et al., 2022; Pont-Fernandez et al., 2022), Butchers broom (Masullo et al., 2016; Rodrigues et al., 2021) Dandelion Root (Ren et al., 2022; Talapphet et al., 2021) and Pleurisy Root (Warashina and Noro, 2010), the plants microbiome has not been investigated in the way that would be most appropriate. Further study into relevance of the microbiome of plant will likely unveil it to be a most significant influencer of immune system response.

## 6. Important considerations

### 6.1. Microbiome presence in botanical medicinal research; false artifact

When conducting experiments in *vitro* on botanical bioactive products, a plant’s anti-inflammatory phytochemicals could be masked as false negatives by an overwhelming presence of MAMPs/LPS. Furthermore, the majority of research carried out on plant extracts do not account for the potential interfering variable comprising the plants inactive microbiome, where MAMPS could reside in consirable concentrations, having amphipathic properties, some heat- and pH-stable. These substances are rarely, validated, tested for, or removed during studies investigating physiological effects of plant medicines. This is not the case for phytochemicals, which are often manufactured for cell culture in endotoxin free form. In other words, it would be necessary to completely remove the plant’s microbiome in order to demonstrate that the plant is operating on a biological target of its own merit.

### 6.2. Chronic inflammation

A second important issue involves possible consequences of routine daily ingestion/ exposure to MAMP laden products. . Long-term use of MAMP-containing goods may cause chronic inflammation, which may fatigue the immune system, upregulate checkpoints, and increase the body’s ability to start carcinogen-mediated carcinogenesis. (Di et al., 2021; Li et al., 2015; Liu et al., 2021). Chronic inflammation, on the other hand, is the root cause of a wide range of human diseases, including auto-immune disorders, osteoporosis, and degenerative joint disease (Mai et al., 2013). These diseases can be significantly reduced by consuming anti-inflammatory plant phytochemicals on a daily basis, which are of great value (Chen et al., 2018; Coutinho-Wolino et al., 2022). Because of this, a lot more research will be needed to understand the long-term health effects of ingesting oral MAMP products, including beta-glucans or probiotics and over an extended period of time.

## 7. Conclusion

There exists a lack of festidious research efforts on the immune-modulating edible plant based microbiome. As a result, this work serves as a pilot study to offer initial insights and provide direction for future research projects that aim to investigate the relevance of the herbal microbiome as a separate entity of functional foods, which influences innate and acquired immunity as a means of “immune boosting”. Further exploration is required in this area some including the following goals; 1) A larger investigation involving recording thousands of products,and comparisons as to plant’s MOC taxonomy according to its place of cultivated origin 2) To establish a rapid valid methodology to identify structural motifes in MAMP antigens to identify pre-existing live taxonomic species associated with those molecules 3) To ascertain the biological effects of main classes of individual re-cultured bioactive strains that have been inactivated and how these act on human micro-biome signaling in the gut and, at what concentrations and 4) To continue forward tracing and identification of bioactive microbial communities during all stages of plant growth from field to harvesting, collections, storage prior to food processing, and point of sale with regard to immune activation.

Additionally, research should be concentrated on figuring out how the major classes of individual re-cultured bioactive strains, both inactivated and activated, affect the human gut microbiome in terms of signaling, concentration, and their use to treat various pathological conditions. The ultimate objective is to determine whether these bioactive strains have the potential to be used as ingestible immunemodulating antigenic-based supplements or medications that can affect long-term acquired immunity in humans. Without the necessity for the plant to serve as an inert reservoir, it could be conceivable to nurture and develop these microorganisms in a bioreactor, kill them, and use antigenic-based supplements or medications to modify human innate and acquired immunity.

## Figures and Tables

**Fig. 5A.**
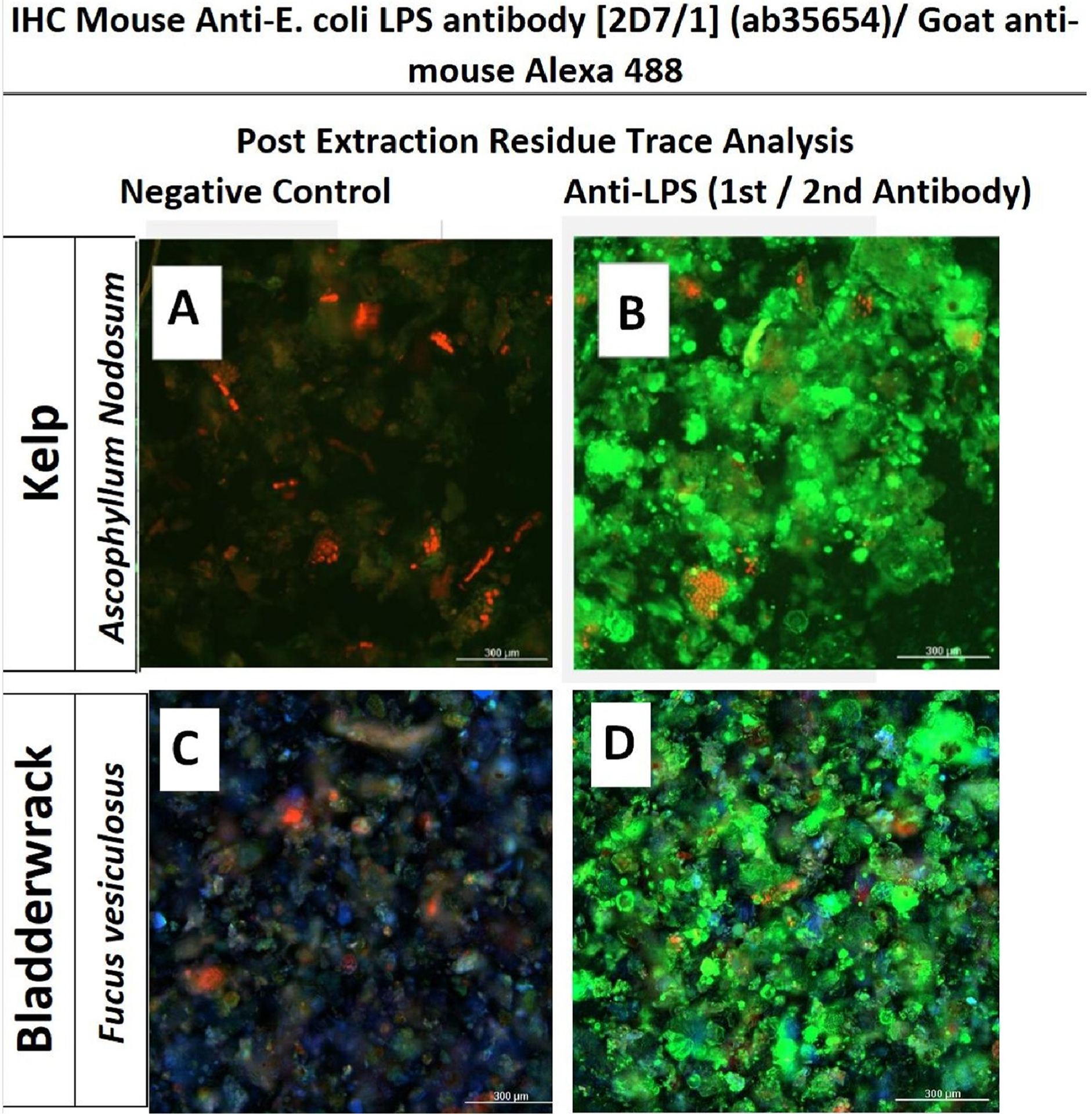
Centrifuge immunohistochemistry of gram-negative cell wall in kelp (Ascophyllum Nodosum) and bladderwrack (Fucus vesiculousus). The images represent auto fluorescence + cross-reactivity with the secondary antibody (goat anti-mouse Alexa 488) (A) kelp (C) bladderwrack (negative controls), with the addition of primary (mouse anti-LPS) (B) kelp (D) bladderwrack. Images were acquired using a digital imaging system, merging red, green, and blue (RGB) images.

**Fig. 5B.**
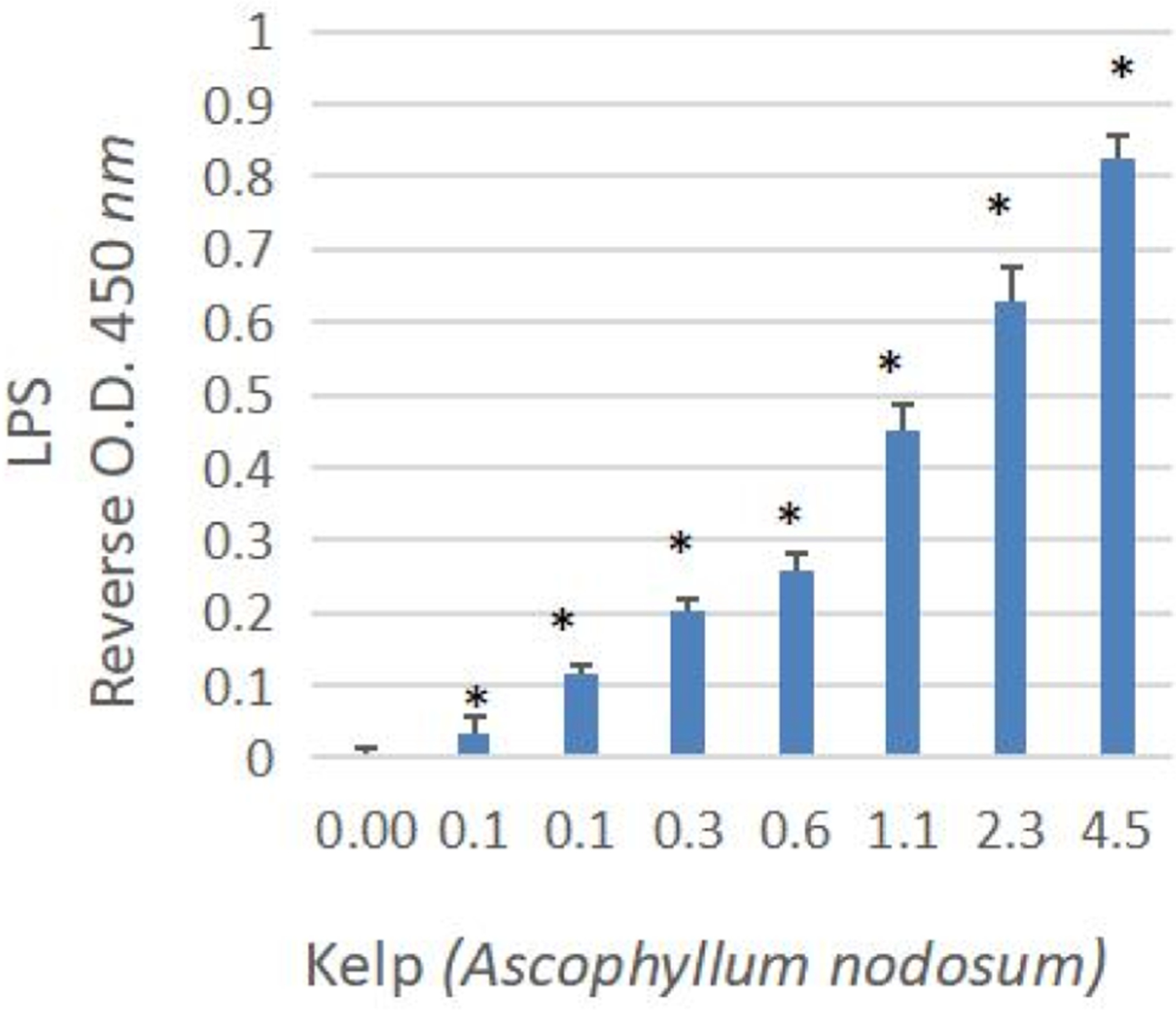
Capture antibody competitive ELISA of LPS in OTC Kelp prepared in sterile endotoxin free PBS. The data represent LPS concentration as reversed O. D. 450 nm and are expressed as the Mean +- S. E. M, n = 3. * Significance from the control using a one-way ANOVA p < 0.05.

**Table 1:** 65 Lead hits presented by Class (“Plant Category”) Common/ Chinese name and Botanical name (“Description”) cross verified by multiple vendors, lot numbers and of various year of issue (“Multiple Vendors”), creating a hit ratio. Pro-inflammatory 50 (PI50)s were determined from a dose response curve (over 10 concentrations.72–371 ug/mL) of the group representative sample (“Rep ID”) by assessing NO production vs. LPS % Control, n = 3 (“Macrophage Activation”). The lower the value the more potent the botanical, and this value is the most relevant. Gram negative cell wall concentrations (Herbal micro-biome) (tested by LAL) are provided as Endotoxin Units (E.U.) where 1 E.U. = 0.5 ng/ml, and reported as E.U./mg and standard capsules sold OTC. Values were then extrapolated to μg/500 mg, for depicting how many μg of pure LPS would be consumed in a 500 mg OTC sold capsule. Beta glucans in herbal extracts were determined and reported as ng/mg (1–3,1–4) lineage and g/100 g for 1–3,1–6 lineage (yeast mushroom), n = 2.

	BOTANICAL IDENTITY			VENDOR HIT RATIO			BIOLOGICAL	HERB micro-biome	HERB micro-biome	HERB micro-biome	HERB micro-biome	HERB micro-biome	
Rep ID	Class	Common Name: Chines/English	Botanical Name/Pharmaceut ical Latin	#Hits	#Vendors	Hit Ratio	Pro-inflammatory (PI50(μg/ml))	E.U./mg	E.U./500 mg	up LPS per 500 mg capsule	β-glucan 1–3,1–4	α-Glucan (g/100g)	β-glucan1–3,1–6 (g/100g)
**AB192**	Root	Bai Tou Weng: English Pulsatil la Root	Pulsatillae Radix	3	3	100%	26.3	1,288	643,925	322	ND	ND	ND
**AB197**	Grass/Straw	Ban Bain Lian: English Chinese Lobelia	Lobelia chinensis herb	2	2	100%	105.3	195	97,636	49	ND	ND	ND
**AB266**	Root	Black Cohosh Root	Cimicifuga racemosa	5	6	83%	109.7	271	135,624	68	ND	1.3	8.4
**AB413**	Hull/Bark/Pods	Black Haw Bark	Viburnum prunifolium	5	5	100%	118.0	219	109,290	55	ND	ND	ND
**AB309**	Hull/Bark/Pods	Black Walnut Hull	Juglans nigra	5	5	100%	6.7	5,000	2,499,961	1,250	ND	0.0	8.8
**AB289**	Sea Algae/Weed	Bladde rwrack	Fucus vesiculosus	6	6	100%	12.6	6,374	3,186,900	1,593	ND	ND	ND
**AB55**	Leaf/Stem	Borage	Borago officinalis	2	2	100%	51.7	509	254,638	127	ND	ND	ND
**AB311**	Root	Burdoc kRoot	Arctium lappa	9	9	100%	9.6	10,748	5,373,844	2,687	ND	0.9	8.0
**AB380**	Root	Butchers Broom Root	Ruscus aculeatus	8	8	100%	14.4	3,162	1,580,995	790	ND	ND	ND
**AB337**	Root	Calamus Root	Acorus calamus	6	8	75%	23.6	391	195,685	98	7.59	0.7	10.5
**AB221**	Root	Cao Wu (Zhi): English Wild Aconit e Root	Aconitum kusnezoffii root prepared	2	2	100%	17.8	172	86,121	43	ND	ND	ND
**AB317**	Fruit/Seed	Cardomumseed	Elettariacardamomum	4	4	100%	31.9	509	254,638	127	ND	0.6	6.0
**AB465**	Mushroom	Chanterelies	Cantharelluscibarius	2	2	100%	45.4	638	318,889	159	ND	0.0	6.7
**AB272**	Grass/Straw	Chickweed	Stellariamedia	3	3	100%	46.0	242	121,074	61	ND	ND	ND
**AB450**	Sea Algae/Weed	Chlorella	Chlorellavulgaris	4	4	100%	13.4	19,811	9,905,722	4,953	ND	0.0	8.9
**AB267**	Grass/Straw	Cleavers	Galiumaparine	6	6	100%	11.6	37,601	18,800,621	9,400	4.8	0.8	5.3
**AB263**	Grass/Straw	Comsilk	Zea mays	5	5	100%	31.3	195	97,636	49	ND	0.9	5.0
**AB281**	Root	Dandelion Root	Taraxacumofficinale	6	8	75%	124.5	257	128,429	64	ND	0.8	5.2
**ab468**	Grass/Straw	Dog Grass Root	Triticumrepens	1	1	100%	44.7	195	97,636	49	ND	ND	ND
**AB156**	Sea Algae/Weed	Dulse Powder	Palmariapalmata	3	3	100%	235.5	172	86,121	43	ND	ND	ND
**AB304**	Root	Echinacea Root	Echinaceapurpurea	6	6	100%	27.4	855	427,353	214	ND	0.8	7.2
**AB236**	Root	Feng Fang: English Saposhnikoviae Root	Radix Saposhnikoviae	3	3	100%	112.2	3,162	1,580,995	790	ND	0.0	6.0
**AB183**	Grass/Straw	Fu Ping/Duckweed	Spirodelapolyrrhizaherb	2	2	100%	31.2	5,197	2,598,318	1,299	ND	0.7	2.3
**AB260**	Leaf/Stem	Homy Goat Weed	Epimediumsagittatum	3	4	75%	45.4	1,089	544,460	272	ND	0.5	0.8
**AB291**	Root	Hydrangea Root	Hydrangeaarborescens	5	5	100%	9.9	855	427,353	214	ND	0.8	6.1
**AB421**	**Sea Algae/Weed**	**Irish Moss**	**Chondruscrispus**	3	4	75%	5.4	7,508	3,754,187	1,877	ND	ND	ND
**AB386**	Root	Kava Kava Root	Pipermethysticum	7	7	100%	33.5	1,824	911,925	456	ND	0.0	10.3
**AB387**	**Sea Algae/Weed**	**Kelp Powder**	**Ascophyllu m Nodosum**	3	5	60%	4.4	53,423	26,711,363	13,356	ND	ND	ND
**AB268**	Fruit/Seed	Kola Nut	Colaacuminata	4	4	100%	49.5	339	169,409	85	ND	0.5	17.6
**AB188**	Root	Lou Lu/Rhaponticum Root	Rhaponticum Uniflorum Root	2	2	100%	14.8	509	254,638	127	ND	ND	ND
**AB216**	Herb	Ma Bian Cao / Verban a	Herba Verbenae	2	2	100%	43.1	242	121,074	61	ND	0.5	2.8
**AB189**	Fruit/Seed	Ma Bo/ Puffbal 1	Calvatia Gigantia Sporophore	2	2	100%	23.8	3,959	1,979,413	990	ND	ND	ND
**AB445**	Grass/Straw	Mai Ya Fructus Hordei Germin atus	Hordeum vulgare fruit	2	2	100%	30.1	132	65,795	33	ND	0.4	2.9
**AB431**	Fruit/Seed	Milk Thistle	Silybummarianum	4	6	67%	33.7	638	318,889	159	ND	0.6	4.9
**AB469**	Bark	Muira Puama Bark Powder	Crotonechioides	3	3	100%	86.8	81	40,448	20	ND	0.6	3.8
**AB408**	Root	Nettle Root	Urtica dioica	5	5	100%	25.2	638	318,889	159	ND	ND	ND
**EM497**	Rice Bran	Orzya Sativa (RiceRoot) Nuo Dao Gen	Orzya Sativa (Rice)	2	2	100%	22.4	1,089	544,460	272	ND	ND	ND
**AB10**	Rice Bran	Orzya Sativa BRM4	Orzya Sativa (Rice)	5	5	100%	334.4	132	65,795	33	11.2	0.5	47.6
**AB316**	Hull/Bark/Pods	Paprika	Capsicumannuum	7	8	88%	123.4	150	74,752	37	ND	ND	ND
**AB366**	Leaf/Stem	Patchouli	Pogostemoncablin	5	5	100%	29.1	242	121,074	61	ND	ND	ND
**AB4**	Leaf’ Stem	Plantain Leaf	Plantago Major	4	4	100%	76.1	105	52,509	26	ND	0.8	2.4
**AB116**	Root	Pleuris y Root	Asclepiastuberosa	2	2	100%	18.4	741	370,323	185	3.74	0.0	3.8
**AB312**	Root	Poke Root	Phytolaccaamericana	3	3	100%	6.2	9,701	4,850,647	2,425	ND	0.8	3.9
**AB213**	Pollen	**Pu Huang/Pollen**	**Pollen Typhae**	2	2	100%	9.0	16,848	8,423,818	4,212	ND	0.0	3.2
**AB214**	Root	Qian Cao/Madder Root	Radix Rubiae	2	2	100%	14.0	314	157,154	79	ND	ND	ND
**AB388**	Root	Saspari 11a	Smilaxmedica	4	4	100%	5.7	2,746	1,372,898	686	2.64	0.5	5.8
**AB78**	Sea Algae/Weed	Seamoss	Irish Moss (Chondruscrispus), Bladderwrack (Fucusvesiculosus), Burdock Root (Arcitumlappa)	1	1	100%	16.6	6,904	3,452,111	1,726	9.69	0.0	11.1
**AB237**	Fruit/Seed	She Chuang Zi	Cnidiummonnierifruit	2	2	100%	31.3	266	133,215	67	ND	ND	ND
**AB180**	Root	Sheng Jiang Pi	Zingiber Officinale Rhizome-Peel	2	2	100%	9.1	290	145,011	73	ND	ND	ND
**AB354**	Root	Soapw ort root	Saponasiaofficinalis	2	2	100%	48.9	1,824	911,925	456	ND	0.0	2.5
**AB258**	Microbe	Spirulina	Arthrospiraplatensis	10	10	100%	17.9	1,089	544,460	272	ND	ND	ND
**AB245**	Root	**Stinging Nettle**	**Urticadioica**	4	4	100%	27.1	6,295	3,147,325	1,574	ND	0.0	2.9
**AB399**	Root	Suma Root	Pfaffiapaniculata	4	4	100%	25.9	150	74,752	37	ND	ND	ND
**AB294**	Fruit/Seed	Tribulu s Fruit	Tribulusterrestris	3	3	100%	17.9	2,746	1,372,898	686	ND	ND	ND
**AB124**	Mushroom	Turkeytailmushroom	Trametesversicolor	3	3	100%	33.0	290	145,011	73	ND	0.0	4.4
**AB271**	herb	Watercress	Nasturtiumofficinale	4	4	100%	18.5	509	254,638	127	ND	ND	ND
**AB334**	Herb	Wormwood Herb	Artemesia Absinthium	4	4	100%	38.2	257	128,429	64	ND	ND	ND
**AB184**	Spike	XiaKu Cao/Heal All	Prunellavulgarisspike	3	3	100%	24.7	195	97,636	49	ND	ND	ND
**AB262**	Hull/Bark/Pods	Yohimbe Bark	Pausinystaliajohimbe	4	5	80%	141.5	290	145,011	73	ND	0.0	9.9
**AB466**	Root	Yucca Root	Yuccaschidigera	5	5	100%	41.5	257	128,429	64	ND	0.0	16.9
**AB205**	Bulb	Zhe Bei Mu	Fritillariathunbergiibulb	2	2	100%	116.8	127	63,543	32	ND	0.0	10.5
**AB446**	Mushroom	Zhu Ling	Polyporusumbellatusfungus	2	2	100%	53.3	314	157,154	79	ND	0.0	9.9
**AB193**	Flower	Zi Hua Di Ding	Violayedoensisherb	2	2	100%	75.1	391	195,685	98	ND	ND	ND
**AB207**	Sea Algae/Weed	Zi Wan	Aster Tataricus root and Rhizome	2	2	100%	35.5	143	71,582	36	ND	ND	ND

**Table 2:** Evaluation of post -column eluents on TRL4 macrophage activation. The data represent Pre (100 %) vs. Post (% of Pre) for macrophage activation at 170 μg/mL for all herbs. The data are expressed as the Mean ± S.D. n = 2 columns/ endotoxin removal. All effluents were tested using the LAL (limulus amebocyte lysate) testing, # denotes positive presence of LPS in post column effluent.

Common Name: Chinese/English	Botanical Name/ Pharmaceutical Latin	Post/Pre % Macrophage Activation	Result
Bai Tou Weng: English Pulsatilla Root	Pulsatillae Radix	1.6 % ± 0.1 %	No Activity
Ban Bain Lian: English Chinese Lobelia	Lobelia chinensis herb	0.2 % ± 0.1 %	No Activity
Black Cohosh Root	Cimicifuga racemosa	0.5 % ± 0.1 %	No Activity
Black Haw Bark	Viburnum prunifolium	0.2 % ± 0.1 %	No Activity
Black Walnut Hull	Juglans nigra	83.4 % ± 4.2 %	Activity #
Bladderwrack	Fucus vesiculosus	95.3 % ± 2.3 %	Activity #
Borage	Borago officinalis	2.5 % ± 0.5 %	No Activity
Burdock Root	Arctium lappa	0.9 % ± 0.5 %	No Activity
Butchers Broom Root	Ruscus aculeatus	2.4 % ± 0.5 %	No Activity
Calamus Root	Acorus calamus	2.3 % ± 0.5 %	No Activity
Cao Wu (Zhi): English Wild Aconite Root	Aconitum kusnezoffii root prepared	0.3 % ± 0.5 %	No Activity
Cardomum seed	Elettaria cardamomum	2.3 % ± 0.8 %	No Activity
Chanterelles	Cantharellus cibarius	2.0 % ± 1.0 %	No Activity
Chickweed	Stellaria media	5.9 % ± 1.0 %	No Activity
Chlorella	Chlorella vulgaris	61.0 % ± 4.5 %	Moderate Activity #
Cleavers	Galium aparine	2.1 % ± 0.8 %	No Activity
Cornsilk	Zea mays	2.2 % ± 0.9 %	No Activity
Dandelion Root	Taraxacum officinale	2.7 % ± 0.9 %	No Activity
Dog Grass Root	Triticum repens	3.1 % ± 0.9 %	No Activity
Dulse Powder	Palmaria palmata	3.1 % ± 1.0 %	No Activity
Echinacea Root	Echinacea purpurea	1.9 % ± 0.8 %	No Activity
Feng Fang: English Saposhnikoviae Root	Radix Saposhnikoviae	2.4 % ± 1.0 %	No Activity
Fu Ping/Duckweed	Spirodela polyrrhiza herb	1.3 % ± 0.9 %	No Activity
Horny Goat Weed	Epimedium sagittatum	4.1 % ± 0.9 %	No Activity
Hydrangea Root	Hydrangea arborescens	1.2 % ± 0.8 %	No Activity
Irish Moss	Chondrus crispus	2.9 % ± 1.0 %	No Activity
Kava Kava Root	Piper methysticum	1.0 % ± 1.1 %	No Activity
Kelp Powder	Ascophyllum Nodosum	98.4 % ± 3.2 %	Activity #
Kola Nut	Cola acuminata	2.4 % ± 1.0 %	No Activity
Lou Lu/Rhaponticum Root	Rhaponticum Uniflorum Root	17.9 % ± 0.8 %	Weak Activity
Ma Bian Cao / Verbana	Herba Verbenae	6.8 % ± 0.6 %	No Activity
Ma Bo/ Puffball	Calvatia Gigantia Sporophore	18.4 % ± 0.3 %	Weak Activity
Mai Ya Fructus Hordei Germinatus	Hordeum vulgare fruit	2.4 % ± 0.7 %	No Activity
Milk Thistle	Silybum marianum	2.2 % ± 0.7 %	No Activity
Muira Puama Bark Powder	Croton echioides	1.3 % ± 0.7 %	No Activity
Nettle Root	Urtica dioica	3.0 % ± 0.6 %	No Activity
Orzya Sativa (Rice Root) Nuo Dao Gen	Orzya Sativa (Rice)	1.8 % ± 0.6 %	No Activity
Orzya Sativa BRM4	Orzya Sativa (Rice)	0.7 % ± 0.8 %	No Activity
Paprika	Capsicum annuum	1.6 % ± 0.8 %	No Activity
Patchouli	Pogostemon cablin	2.2 % ± 0.7 %	No Activity
Plantain Leaf	Plantago Major	2.9 % ± 0.8 %	No Activity
Pleurisy Root	Asclepias tuberosa	0.0 % ± 0.8 %	No Activity
Poke Root	Phytolacca americana	0.0 % ± 0.9 %	No Activity
Pu Huang /Pollen	Pollen Typhae	97.3 % ± 0.4 %	Activity #
Qian Cao/Madder Root	Radix Rubiae	0.4 % ± 0.9 %	No Activity
Sarsaparilla Root	Smilax medica	2.4 % ± 1.0 %	No Activity
Seamoss	Combination: Irish Moss (Chondrus crispus), Bladderwrack (Fucus vesiculosus), Burdock Root (Arcitum lappa)	93.7 % ± 2.7 %	Activity #
She Chuang Zi	Cnidium monnieri fruit	3.5 % ± 1.2 %	No Activity
Sheng Jiang Pi	Zingiber Officinale Rhizome-Peel	4.8 % ± 0.9 %	No Activity
Soapwort root	Saponasia officinalis	0.0 % ± 1.1 %	No Activity
Spirulina	Arthrospira platensis	22.8 % ± 1.2 %	Weak Activity #
Stinging Nettle	Urtica dioica	6.5 % ± 0.9 %	No Activity
Suma Root	Pfaffia paniculata	0.5 % ± 1.2 %	No Activity
Tribulus Fruit	Tribulus terrestris	0.3 % ± 0.7 %	No Activity
Turkey tail mushroom	Trametes versicolor	2.6 % ± 0.9 %	No Activity
Watercress	Nasturtium officinale	0.9 % ± 1.0 %	No Activity
Wormwood Herb	Artemesia Absinthium	1.4 % ± 1.0 %	No Activity
Xia Ku Cao / Heal All	Prunella vulgaris spike	1.3 % ± 0.9 %	No Activity
Yohimbe Bark	Pausinystalia johimbe	1.3 % ± 2.9 %	No Activity
Yucca Root	Yucca schidigera	1.6 % ± 1.2 %	No Activity
Zhe Bei Mu	Fritillaria thunbergii bulb	2.3 % ± 1.2 %	No Activity
Zhu Ling	Polyporus umbellatus fungus	4.6 % ± 1.5 %	No Activity
Zi Hua Di Ding	Viola yedoensis herb	1.9 % ± 1.0 %	No Activity
Zi Wan	Aster Tataricus root	1.5 % ± 1.1 %	No Activity

**Table 3:** Taxonomical identity of reverse cultured OTC/ POS herbal products. Sequencing for both global colonies and gram negative isolation was carried out using ribosomal 18S, ITS1, 5.8 s, ITS2 28S and 16S rDNA sequence (1,542 bp) coding regions for species identification and community analysis. The data is listed by class, taxonomy, herb tested and abundance.

Type	Description	Reverse Culture	Value*	Gram-	Gram-Value**
Gram-negative	s__Klebsiella_aerogenes	Stinging Nettle Root	58.83	POS	54.91
Fungus	s__uncultured_Glomeraceae	Stinging Nettle Root	34.51	NA	14.01
Gram-negative	s	Stinging Nettle Root	29.12	POS	28.37
Fungus	s__uncultured_Ascomycota	Stinging Nettle Root	25.33	NA	2.75
Fungus	s_Cryptococcus_sp._F19–3–1	Stinging Nettle Root	12.00	NA	0.00
Fungus	s__Dothideomycetes_sp._LS-2013 g	Stinging Nettle Root	9.82	NA	0.00
NA	s__unclassified	Stinging Nettle Root	5.20	NA	0.30
Plant	s__Quercus_suber	Stinging Nettle Root	4.16	NA	0.00
Fungus	s__Aureobasidium_pullulans	Stinging Nettle Root	3.73	NA	0.00
Fungus	s__Naganishia_albida	Stinging Nettle Root	2.56	NA	0.00
Gram-negative	s__Gammaproteobacteria_unclassified	Stinging Nettle Root	2.09	POS	0.71
Gram-negative	s__uncultured_Enterobacter_sp.	Stinging Nettle Root	1.30	POS	0.00
Gram-Positive	s__Clostridium_sensu_stricto_18_unclassified	Stinging Nettle Root	1.28	NA	0.02
Fungus	s__Fissuroma_neoaggregatum	Stinging Nettle Root	0.75	NA	0.00
algal eukaryotes	s__Mallomonas_striata	Stinging Nettle Root	0.74	NA	1.29
Gram-negative	s__Enterobacter_sp.	Stinging Nettle Root	0.70	POS	0.44
Gram-negative	s__Enterobacter_hormaechei	Stinging Nettle Root	0.60	POS	0.40
Fungus	s__Sterigmatomyces_halophilus	Stinging Nettle Root	0.57	NA	0.00
Fungus	s__Malassezia_globosa	Stinging Nettle Root	0.55	NA	0.00
Gram-negative	s__Enterobacter_sp._GIST-NKst8	Stinging Nettle Root	0.53	POS	0.32
Gram-negative	s__Atlantibacter_hermannii	Stinging Nettle Root	0.46	POS	0.00
Gram-negative	s__Enterobacter_sp._MZ_1	Stinging Nettle Root	0.37	POS	0.00
Gram-negative	s__Pantoea_septica	Stinging Nettle Root	0.23	POS	0.00
Gram-negative	s__uncultured_Klebsiella_sp.	Stinging Nettle Root	0.18	POS	0.00
Gram-Positive	s__Enterobacteriaceae_unclassified	Stinging Nettle Root	0.16	POS	0.00
Gram-negative	s__Paenibacillus_sp._RAG-53	Stinging Nettle Root	0.16	NA	0.00
Gram-negative	s__Cronobacter_sakazakii	Yucca Root	84.02	POS	76.41
Fungus	s__Irpex_lacteus	Yucca Root	21.09	NA	0.01
NA	s__unclassified	Yucca Root	17.99	NA	33.70
Fungus	s__Chlorociboria_clavula	Yucca Root	16.22	NA	0.00
Fungus	s__uncultured_Ascomycota	Yucca Root	14.58	NA	0.00
Fungus	s__uncultured_Glomeraceae	Yucca Root	8.76	NA	0.01
Fungus	s_Cryptococcus_sp._F19–3–1	Yucca Root	8.61	NA	0.00
Fungus	s__Taphrina_pruni	Yucca Root	6.70	NA	0.00
Yeast	s__Meyerozyma_guilliermondii	Yucca Root	6.17	NA	0.00
Fungus	s__uncultured_Chytridiomycota	Yucca Root	5.88	NA	0.00
Fungus	s__Fusarium_verticillioides	Yucca Root	5.32	NA	0.00
Gram-negative	s__Cronobacter_malonaticus	Yucca Root	2.66	POS	2.50
Plant	s__Quercus_suber	Yucca Root	2.61	NA	0.00
Gram-negative	s__Pantoea_agglomerans	Corn Silk	37.72	POS	46.64
Fungus	s__Aspergillus_sp._JUC-2	Corn Silk	37.41	NA	0.00
Spiromonas	s__Colpodella_angusta	Corn Silk	23.43	NA	0.00
Gram-negative	s__Klebsiella_aerogenes	Corn Silk	20.10	POS	23.60
NA	s__unclassified	Corn Silk	19.19	NA	0.46
Gram-negative	s__Enterobacter_cloacae	Corn Silk	10.06	POS	12.74
Gram-negative	s__Enterobacter_sp._VJ-6	Corn Silk	9.89	POS	12.63
Gram-negative	s__Escherichia_sp._CPD32	Corn Silk	3.70	POS	0.03
Gram-negative	s__uncultured_Enterobacter_sp.	Corn Silk	2.53	POS	0.00
Fungus	s__Penicillium_decumbens	Corn Silk	1.99	NA	0.00
Gram-negative	s__Enterobacter_sp._MZ_1	Corn Silk	1.76	POS	0.00
Fungus	s__Malassezia_restricta	Corn Silk	1.49	NA	0.00
Gram-negative	s__uncultured_Klebsiella_sp.	Corn Silk	1.38	POS	1.53
Gram-negative	s__Enterobacter_sp.	Corn Silk	1.16	POS	0.38
Gram-negative	s__Atlantibacter_hermannii	Corn Silk	1.03	POS	0.00
Gram-negative	s__Gammaproteobacteria_unclassified	Corn Silk	0.97	POS	0.42
Gram-negative	s__Pantoea_septica	Corn Silk	0.65	POS	0.00
Gram-Positive	s__Bacillus_sp._IDA4921	Corn Silk	0.58	NA	0.00
Gram-negative	s__Enterobacter_ludwigii	Corn Silk	0.49	POS	0.61
Gram-negative	s__Kosakonia_cowanii	Corn Silk	0.44	POS	0.00
Gram-negative	s__Enterobacter_sp._enrichment_culture_clone_HSL39	Corn Silk	0.39	POS	0.44
Gram-negative	s__Enterobacter_sp._UIWRF0036	Corn Silk	0.35	POS	0.00
Gram-negative	s__Enterobacteriaceae_unclassified	Corn Silk	0.35	POS	0.00
Gram-Positive	s__Lactobacillus_crispatus	Corn Silk	0.22	NA	0.00
Gram-Positive	s__Enterobacter_asburiae	Corn Silk	0.21	NA	0.31
Type	Description	Reverse culture		Value*	Gram-
Gram-Positive	s__Fontibacillus_unclassified	Black Walnut Hull		44.24	NA
Gram-Positive	s__Paenibacillus_unclassified	Black Walnut Hull		37.04	NA
Fungus	s__Aureobasidium_pullulans	Black Walnut Hull		27.17	NA
Fungus	s__Aspergillus_sp._JUC-2	Black Walnut Hull		21.90	NA
Fungus	s__Malassezia_sympodialis	Black Walnut Hull		13.27	NA
NA	s__unclassified	Black Walnut Hull		11.73	NA
Fungus	s__Irpex_lacteus	Black Walnut Hull		10.12	NA
Gram-Positive	s__Bacillus_sp._IDA4921	Black Walnut Hull		4.94	NA
Fungus	s__Cladosporium_sphaerospermum	Black Walnut Hull		4.31	NA
Gram-negative	s__Anaerospora_unclassified	Black Walnut Hull		3.38	POS
Gram-Positive	s__Bacillus_thermoamylovorans	Black Walnut Hull		2.98	NA
Gram-Positive	s__Geobacillus_thermoleovorans	Black Walnut Hull		1.31	NA
Gram-Positive	s__uncultured_Paenibacillus_sp.	Black Walnut Hull		1.00	NA
Gram-Positive	s__uncultured_Bacillus_sp.	Black Walnut Hull		0.81	NA
Gram-Positive	s__Paenibacillus_yonginensis	Black Walnut Hull		0.57	NA
Gram-Positive	s__Fontibacillus_phaseoli	Black Walnut Hull		0.36	NA
Gram-Positive	s__Enterococcus_gallinarum	Burdock_Root		73.12	NA
Fungus	s__uncultured_Glomeraceae	Burdock_Root		46.41	NA
Fungus	s__Fibroporia_albicans	Burdock_Root		21.61	NA
Yeast	s__Rhodotorula_sp._CH4	Burdock_Root		17.85	NA
Mite	s__Demodex_folliculorum	Burdock_Root		9.66	NA
Gram-Positive	s__Bacillus_sp._IDA4921	Burdock_Root		4.06	NA
Gram-Positive	s__Enterococcus_faecium	Burdock_Root		3.51	NA
Algal eukaryotes	s__Mallomonas_striata	Burdock_Root		1.25	NA
Gram-Positive	s__bromate-reducing_bacterium_B6	Burdock_Root		1.15	NA
Gram-negative	s__Bilophila_unclassified	Burdock_Root		0.95	POS
Gram-Positive	s__Pediococcus_acidilactici	Burdock_Root		0.74	NA
Gram-Positive	s__Bacillus_proteolyticus	Burdock_Root		0.36	NA
Gram-Positive	s__uncultured_Enterococcus_sp.	Burdock_Root		0.20	NA
Gram-Positive	s__Enterococcus_sp._MA3	Burdock_Root		0.17	NA
NA	s__unclassified	Echinacea Root		37.34	NA
Fungus	s__uncultured_Glomeraceae	Echinacea Root		36.53	NA
Gram-Positive	s__bromate-reducing_bacterium_B6	Echinacea Root		18.76	NA
Gram-Positive	s__Clostridium_sensu_stricto_7_unclassified	Echinacea Root		12.50	NA
Gram-Positive	s__Lachnotalea_unclassified	Echinacea Root		7.78	NA
Gram-Positive	s__Lysinibacillus_unclassified	Echinacea Root		7.59	NA
Gram-Positive	s__Clostridium_sensu_stricto_18_unclassified	Echinacea Root		7.34	NA
Fungus	s__Bjerkandera_sp._CPCC_480726	Echinacea Root		7.33	NA
Gram-Positive	s__Clostridium_sensu_stricto_3_unclassified	Echinacea Root		7.03	NA
Fungus	s__Malassezia_sympodialis	Echinacea Root		6.80	NA
Gram-Positive	s__Clostridium_sp._K7	Echinacea Root		3.84	NA
Fungus	s__Aspergillus_sp._JUC-2	Echinacea Root		3.83	NA
Plant	s__Quercus_suber	Echinacea Root		3.66	NA
Gram-Positive	s_Eubacterium]_fissicatena_group_unclassified	Echinacea Root		2.93	NA
Gram-Positive	s__Clostridium_sensu_stricto_1_unclassified	Echinacea Root		2.73	NA
Fungus	s__Chlorociboria_clavula	Echinacea Root		2.54	NA
Gram-Positive	s__Clostridiaceae_unclassified	Echinacea Root		2.14	NA
Gram-Positive	s__Clostridium_sp._BTY5	Echinacea Root		1.82	NA
Gram-Positive	s__uncultured_Bacillus_sp.	Echinacea Root		1.42	NA
Probiotic Bacteria	s__Lachnospiraceae_unclassified	Echinacea Root		1.26	NA
Probiotic Bacteria	s__Caproiciproducens_unclassified	Echinacea Root		1.26	NA
Gram-Positive	s__Clostridium_sp._Iso-A2	Echinacea Root		0.99	NA
Probiotic Bacteria	s__Faecalicatena_contorta	Echinacea Root		0.89	NA
Gram-Positive	s__Clostridium]_amygdalinum	Echinacea Root		0.61	NA
Gram-Positive	s__Clostridium_sensu_stricto_10_unclassified	Echinacea Root		0.51	NA
Gram-Positive	s__Clostridium]_xylanolyticum	Echinacea Root		0.48	NA
Gram-Positive	s__Bacillus_cereus	Echinacea Root		0.38	NA
Gram-Positive	s__Paenibacillus_unclassified	Kava Kava Root		50.23	NA
Fungus	s__uncultured_Glomeraceae	Kava Kava Root		49.20	NA
Fungus	s__Fibroporia_albicans	Kava Kava Root		23.00	NA
Gram-Positive	s__Bacillus_thermoamylovorans	Kava Kava Root		16.59	NA
Fungus	s__Malassezia_restricta	Kava Kava Root		16.23	NA
Gram-Positive	s__Fontibacillus_unclassified	Kava Kava Root		10.56	NA
Fungus	s__Auricularia_polytricha	Kava Kava Root		8.07	NA
Fungus	s__Chlorociboria_clavula	Kava Kava Root		6.59	NA
Gram-Positive	s__uncultured_Bacillus_sp.	Kava Kava Root		4.35	NA
Gram-Positive	s__Geobacillus_thermoleovorans	Kava Kava Root		2.42	NA
single cell eukaryotes	s__Colpoda_steinii	Kava Kava Root		2.40	NA
Yeast	s__Meyerozyma_guilliermondii	Kava Kava Root		2.39	NA
Gram-Positive	s__bromate-reducing_bacterium_B6	Kava Kava Root		2.37	NA
Gram-Positive	s__Bacillus_sp._IDA4921	Kava Kava Root		0.96	NA
Gram-Positive	s__Bacillus_sp._SAB	Kava Kava Root		0.88	NA
plant	s__Ginkgo_biloba	Kava Kava Root		0.86	NA
Gram-Positive	s__Paenibacillus_sp.	Kava Kava Root		0.80	NA
Gram-Positive	s__Enterococcus_faecium	Kava Kava Root		0.22	NA
Gram-Positive	s__Ligilactobacillus_unclassified	Kava Kava Root		0.21	NA
Gram-Positive	s__Clostridium_sp._KOPRI80182	Seamoss		51.95	NA
NA	s__unclassified	Seamoss		33.95	NA
Fungus	s__uncultured_Glomeraceae	Seamoss		33.80	NA
Gram-Positive	s__Clostridium_sensu_stricto_1_unclassified	Seamoss		19.46	NA
Fungus	s__Aspergillus_sp._JUC-2	Seamoss		11.57	NA
Gram-Positive	s__bromate-reducing_bacterium_B6	Seamoss		7.65	NA
Fungus	s__Bjerkandera_sp._CPCC_480726	Seamoss		6.22	NA
Fungus	s__Cryptococcus_sp._F19–3–1	Seamoss		4.54	NA
Gram-Positive	s__Bacillus_thermoamylovorans	Seamoss		4.36	NA
Gram-Positive	s__Clostridium_sensu_stricto_18_unclassified	Seamoss		3.53	NA
Fungus	s__Ceriporiopsis_sp._s12–2	Seamoss		3.40	NA
Gram-Positive	s__Clostridium_sensu_stricto_12_unclassified	Seamoss		2.47	NA
Gram-Positive	s__uncultured_Bacillus_sp.	Seamoss		2.00	NA
Gram-Positive	s__Clostridium_sp._JC242	Seamoss		0.97	NA
Gram-Positive	s__Bacillus_sp._CanS-96	Seamoss		0.73	NA
Gram-Positive	s__Bacillus_cereus	Seamoss		0.66	NA
Gram-Positive	s__Bacillus_sp._A17	Seamoss		0.65	NA
Gram-Positive	s__Clostridium]_amygdalinum	Seamoss		0.21	NA

## Data Availability

No data was used for the research described in the article.

## Abbreviations:

APC: antigen presenting cells
ASV: amplicon sequence variants
CCL: (C-C motif) ligand
CFU: colony forming units
CXCL: chemokine (C-X-C motif) ligand
DADA2: divisive amplicon denoising algorithm
DMEM: dulbecco’s modified eagle medium
DMSO: dimethyl Sulfoxide
GALT: gut-associated lymphoid tissue
HBSS: hank’s balance salt solution
HCl: hydrochloric acid
HEPES: N-2-hydroxyethylpiperazine-N-2-ethane sulfonic acid
HTS: high throughput screening
IL: interleukin
iNOS: inducible nitric oxide synthase
LAL: limulus amebocyte lysate
LPS: lipopolysaccharide
MAMPs: microbial associated molecular pattern
MyD88: myeloid differentiation primary response gene 88
NaOh: sodium hydroxide
NK: natural killer
NO2–: nitric oxide
OTC: over the counter
PAMPs: pathogen associated molecular patterns
PBS: phosphate buffered saline
POLYIC: polyinosinic-polycytidylic acid
POS: point of sale
PRR: pathogen/pattern recognition receptors
TAM: tumor associated macrophage
TLR: toll-like receptor
TNF: tumor necrosis factor
WHO: World Health Organization
